# Monoamine-induced diacylglycerol signaling rapidly accumulates Unc13 in nanoclusters for fast presynaptic potentiation

**DOI:** 10.1073/pnas.2514151122

**Published:** 2025-08-20

**Authors:** Natalie Blaum, Tina Ghelani, Torsten W. B. Götz, Keagan S. Chronister, Mercedes Bengochea, Livia Ceresnova, Christian F. Christensen, Thiago C. Moulin, Hanna Kern, Ulrich Thomas, Martin Heine, Stephan J. Sigrist, Alexander M. Walter

**Affiliations:** ^a^Molecular and Theoretical Neuroscience, Department of Neuroscience, Faculty of Health and Medical Sciences, University of Copenhagen, Copenhagen 2200, Denmark; ^b^Molecular and Theoretical Neuroscience, Leibniz-Forschungsinstitut für Molekulare Pharmakologie, Berlin 13125, Germany; ^c^Institute for Biology and Genetics, Freie Universität Berlin, Berlin 14195, Germany; ^d^Functional Neurobiology, Institute of Developmental Biology and Neurobiology, Johannes Gutenberg University Mainz, Mainz 55128, Germany; ^e^Department of Cellular Neurobiology, Leibniz Institute for Neurobiology, Magdeburg 39118, Germany

**Keywords:** synaptic plasticity, neurotransmitter release, Munc13, Neuromodulation, G-protein- coupled receptors

## Abstract

Neuromodulators control mood, arousal, and behavior by inducing synaptic plasticity via G-protein-coupled receptors. While long-term presynaptic potentiation requires structural changes, mechanisms enabling potentiation within minutes remain unclear. Using the *Drosophila* neuromuscular junction, we show that octopamine, the invertebrate analog of norepinephrine, potentiates evoked neurotransmitter release on the timescale of one minute via a G-protein-coupled pathway involving presynaptic OAMB receptors and phospholipase C. This fast potentiation correlates with elevated signals of the release factor Unc13A and the scaffolding protein Bruchpilot. Live, single-molecule imaging of endogenously tagged Unc13 revealed its instantly reduced motility and increased concentration in synaptic nanoclusters with potentiation. Presynaptic knockdown of Unc13A fully blocked fast potentiation. Moreover, deleting its N-terminal localization sequence mislocalized the protein fragment to the cytosol, but still allowed for rapid plasma membrane recruitment by diacylglycerol (DAG) analog phorbol esters and octopamine, implicating a role of more C-terminal domains. A point mutation of endogenous Unc13 in its DAG-binding C1 domain blocked plasticity-induced nanoscopic enrichment and synaptic potentiation. The mutation increased basal neurotransmission but reduced Unc13 levels, revealing a gain of function and potential homeostatic compensation. The mutation also blocked phorbol ester–induced potentiation, decreased the calcium sensitivity of neurotransmission, and caused short-term synaptic depression. Homeostatic potentiation induced by postsynaptic receptor block mirrored octopamine-induced Unc13 recruitment and required presynaptic OAMB receptors, indicating overlapping machinery. Thus, rapid Unc13 immobilization and nanoscale compaction are salient features of fast presynaptic potentiation.

## Significance

Neuromodulators rapidly modulate synaptic transmission, yet the pathways linking them to presynaptic release machinery remain largely unknown. Understanding how monoamines intersect with synaptic targets is important for deciphering brain function. Here, we define a pathway for fast synaptic potentiation: from the monoamine octopamine, its receptor, and signaling lipid [diacylglycerol (DAG)] to its effector, (M)Unc13, driving its nanoscopic enrichment and reduced motility within minutes. Disrupting (M)Unc13’s DAG sensitivity alters baseline synaptic activity, and blocks octopamine-induced potentiation and the protein’s nanoscopic compaction, suggesting an important role of this domain in fast synaptic modulation. Given the conserved nature of the release machinery and the analogous role of norepinephrine in vertebrates, these findings likely reflect a broader principle of monoaminergic neuromodulation.

Synaptic transmission relies on neurotransmitter release from synaptic vesicles (SVs) to activate postsynaptic receptors. Plasticity of transmission strength is needed for neural processing, signal stabilization, and information storage. Presynaptic plasticity alters neurotransmitter release which relies on a highly conserved molecular machinery to target (dock) SVs to the plasma membrane and to molecularly prime them for fusion with the plasma membrane in response to action potentials (APs). SV docking, priming, and fusion depend on neuronal soluble NSF attachment protein receptor (SNARE) proteins that form a complex between SV- and plasma membranes and on the SNARE-binding proteins (M)Unc13 and (M)Unc18 (“M” in the mammalian case) (1). The SV protein Synaptotagmin triggers SV fusion in response to presynaptic calcium (Ca^2+^) influx through voltage gated ion channels (2). The local topology of subsynaptic active zones (AZs) where SV fusion takes place is controlled by large multidomain cytomatrix proteins like the Rab3 Interacting Molecule (RIM), RIM-Binding Protein (RIM-BP), and Bruchpilot (BRP in *Drosophila melanogaster*) or ELKS/CAST (in mammals) (3, 4).

Long-term potentiation of neurotransmitter release mediates homeostasis and information storage and coincides with structural adaptations of Ca^2+^ channels, RIM, RIM-BP, BRP, and (M)Unc13 (5–11). Among those factors, (M)Unc13 adaptations appear required, pointing to a pivotal role in synaptic plasticity (5). Mechanisms underlying intermittent potentiation on a minutes’ timescale remain unclear but may involve the evolutionarily conserved (M)Unc13 regulatory CaM-binding-, C2B-, and C1-domains which bind Ca^2+^/Calmodulin, Ca^2+^/phosphatidylinositol4,5-bisphosphate (PI(4, 5)P_2_) and diacylglycerol (DAG), respectively (12, 13). Indeed, DAG analog phorbol esters rapidly potentiate neurotransmitter release via the (M)Unc13 C1 domain, though the reasons for this and the biological pathway involved remain elusive (14, 15).

Neuromodulator binding to G-protein-coupled receptors (GPCRs) induces conformational changes leading to the intracellular dissociation of the heteromeric G-protein complex into the Gα and Gβ/γ subunits which modulate downstream effector pathways. Gαi/o and Gαs pathways inhibit or activate adenylate cyclase to generate cyclic-adenosine monophosphate (cAMP) which activates Protein Kinase A and regulates synapse growth, short-term plasticity, long-term potentiation, animal locomotion, and learning and memory (16–21). The Gαq/11 pathway activates phospholipase C (PLC) to hydrolyze PI(4, 5)P_2_ into DAG and Inositol trisphosphate (IP_3_) which in turn activate protein kinase C (PKC) and liberate Ca^2+^ from internal stores. PKC phosphorylation of Ca^2+^ channels, (M)Unc18, SNAP25, and Synaptotagmin modulates neurotransmitter release (22–25) and genetic experiments in **Caenorhabditis* elegans* suggested a connection to (M)Unc13 as well (26). However, whether neuromodulators potentiate neurotransmitter release via direct action of the signaling lipids DAG and PI(4, 5)P_2_ on (M)Unc13 and how this might affect the protein at the synapse remains to be established.

In insects, octopamine, the invertebrate analog of mammalian noradrenaline, is a major neuromodulator of synaptic structure and function, locomotion, and learning and memory (16, 27–31). At the *D. melanogaster* neuromuscular junction (NMJ), acute octopamine application enhances synaptic transmission, elevates cAMP levels, and triggers the release of neuropeptides, while prolonged exposure enhances neural growth (16, 32–35). Octopamine can signal via the Gαq/PLC pathway (34), and here we investigate its role in fast synaptic potentiation via the neurotransmitter release machinery. We combine electrophysiological recordings, GCaMP8f-based presynaptic Ca^2+^ imaging, confocal microscopy, and live single-molecule imaging at the *Drosophila* larval NMJ with genetic and pharmacological manipulations. Our findings point to a requirement of presynaptic OAMB octopamine receptors, PLC and Unc13 for fast potentiation. We provide a mechanism of how this monoamine signaling engages the Unc13 C1 domain to enhance synaptic output and rapidly enrich Unc13 in subsynaptic nanoclusters on a minute’s timescale. Phorbol ester–induced potentiation or presynaptic homeostatic potentiation (PHP) coincided with similar nanoscale Unc13 compaction, suggesting a shared relevance of these subtle structural changes in potentiating neurotransmitter release.

## Results

To identify molecular mechanisms of how neuromodulators rapidly modulate synaptic transmission, we investigated the molecular pathway for acute octopamine signaling at the *D. melanogaster* third-instar larval NMJ.

### Octopamine Treatment Rapidly Increases Neurotransmitter Release and BRP and Unc13 Signals.

**Fig. 1.**
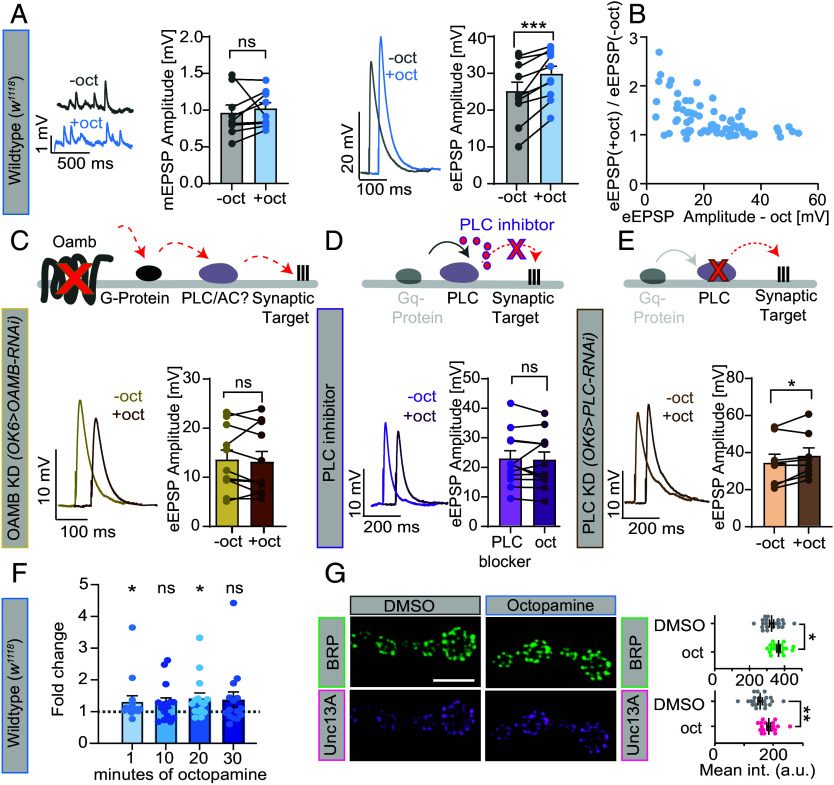
1-min octopamine treatment potentiates neurotransmitter release dependent on OAMB receptors and phospholipase C and rapidly enhances BRP and Unc13A AZ signals. (*A*, *Left*) Representative example traces of spontaneous mEPSPs from wildtype larvae (Ctrl, w1118) before (gray, *Top*) and 1-min after 20 µM octopamine incubation (blue, *Bottom*) together with a cell-wise quantification of mEPSP amplitudes before (−oct, gray) and 1 min after incubation with 20 µM octopamine (+oct, blue). (*Right*) Representative eEPSP responses from control synapses before (gray) and after octopamine incubation (blue) and quantification of eEPSP amplitudes (−oct, gray; +oct, blue). (*B*) Fold-change of eEPSP amplitudes after/before octopamine in control animals as a function of the eEPSP amplitude before treatment (data pooled from experiments with 0.4 mM and 0.2 mM external Ca^2+^). (*C*, *Top*) Illustration OAMB knockdown approach. Below: Representative eEPSP responses following OAMB receptor knockdown (KD) in type I motoneurons (*OK6-Gal4>UAS-OAMB-RNAi*) before (yellow) and 1-min after incubation with 20 µM octopamine (+oct, brown) (for genetic control experiments, please see *SI Appendix*, Fig. S1*G*). (*D*, *Top*) Illustration of PLC inhibition. Below: Representative eEPSP responses from wildtype (w1118) larvae with 1-min PLC inhibitor incubation (U73122, 1 µM; light purple) and followed by 1-min incubation with 20 µM octopamine (dark purple) with quantification of eEPSP amplitudes. For control experiments using a control drug, see *SI Appendix*, Fig. S2*B*. (*E*) Same as (*C*) for PLC knockdown (KD, *OK6-Gal4>UAS-PLC-RNAi*). (*F*) Fold-change of eEPSP amplitudes from wildtype larvae after/before octopamine treatment for 1, 10, 20, and 30 min of octopamine incubation. (*G*, *Left*) Representative confocal images of muscle 4 NMJs from wildtype *Drosophila* larvae (w1118) labeled with antibodies against BRP (green, *Top*) and Unc13A (pink, *Bottom*) either treated for 1 min with Dimethyl Sulfoxide (DMSO) or octopamine in DMSO (20 µM). (*Right*) Quantification of BRP and Unc13A AZ intensities. Current clamp recordings (*A*, *C*, *D*, and *E*) were performed on muscle 6 NMJs in the presence of 0.4 mM extracellular Ca^2+^. Number of cells (n) and animals (N) investigated: n/N(Wildtype, *A*) = 12/12; n/N(Wildtype, *B*) = 70/70; n/N(OAMB KD, *C*) = 11/11; n/N(PLC inhibitor, *D*) = 12/12; n/N(PLC KD, *E*) = 8/8; n/N(Wildtype, *F*) = 15/15 n/N(DMSO, *G*) = 21/7, n/N(octopamine, *G*) = 19/7. For exact genotypes, see *Material and Methods*. Data depict mean values ± SEM. Statistical analysis with paired parametric *t* tests (*A*, *C*, *D*, and *E*), Friedman test (*F*), or unpaired *t* test (*G*). For details, see *SI Appendix*, Table S2. n.s., *P* > 0.05; *P 0.05; **P 0.01; ***P 0.001. [Scale bars: 5 µm (*G*).]

We tested whether acute octopamine treatment affected synaptic transmission in current-clamp recordings from muscle 6 NMJs. We quantified miniature excitatory postsynaptic potentials (mEPSPs), reporting on spontaneous fusion events of individual neurotransmitter-containing SVs; and AP-evoked excitatory postsynaptic potentials (eEPSPs), reporting on synchronized SV fusion at a Ca^2+^ concentration of 0.4 mM (Fig. 1*A*) in the external solution (or 0.2 mM–Fig. 1*F*; 0.3 mM-*SI Appendix*, Fig. S1, 0.1 mM-*SI Appendix*, Fig. S10) to prevent muscle contraction and to ensure near-linear summation of mEPSPs to eEPSPs (36). Exposing larval fillets to 20 µM octopamine for 1 min neither affected mEPSP amplitudes nor frequencies but significantly potentiated eEPSP amplitudes (Fig. 1*A* and *SI Appendix*, Fig. S1 *A*–*C*), consistent with previous reports (16). Potentiation depended on octopamine and not the simulation protocol (*SI Appendix*, Fig. S1*A*) and longer exposure did not potentiate eEPSPs further (Fig. 1*F*).

While the OAMB receptor is primarily known for its role in learning in the central fly brain (31, 37), it was recently shown to be expressed in the motoneurons and to enhance synaptic activity on the timescale of 10 min (34, 38). We therefore investigated its role in fast potentiation by its specific knockdown in motoneurons. While baseline AP-evoked synaptic transmission was unaffected by presynaptic OAMB RNAi expression (OAMB KD, *OK6-Gal4>UAS-OAMB-RNAi*) (*SI Appendix*, Fig. S1*F*), acute octopamine-induced potentiation was blocked (Fig. 1*C* and *SI Appendix*, Fig. S1*H*) (but present in the respective control animals, *SI Appendix*, Fig. S1*G*). We next examined whether octopamine-induced potentiation occurred via the Gαq pathway and therefore depended on PLC. In the presence of the PLC inhibitor U73455 octopamine-induced potentiation of eEPSPs was blocked (Fig. 1*D*), consistent with recent reports (34), while robust potentiation was seen in parallel recordings using a control drug (U73343) (*SI Appendix*, Fig. S2*B*). The drug itself had no detectable effects on mEPSPs or baseline eEPSPs (*SI Appendix*, Fig. S2). RNAi-mediated PLC-knockdown in motoneurons (*OK6-Gal4>UAS-PLC-RNAi*) did not fully block octopamine-induced eEPSP-potentiation but reduced its magnitude compared to control animals (*UAS-PLC-RNAi*, without *OK6-Gal4* driver) (Fig. 1*E* and *SI Appendix*, Fig. S2*H*). Knockdown did not affect baseline eEPSP amplitudes, but increased mEPSP amplitudes (*SI Appendix*, Fig. S2*L*). Collectively, these results indicate that the presynaptic Gαq pathway mediates octopamine-induced potentiation.

Potentiation could be due to increased AP-induced Ca^2+^ levels (39). We therefore performed live-imaging experiments using the fluorescent Ca^2+^ indicator Syt::mScarlet::GCaMP8f (mScar8f) targeted to SVs expressed in motor neurons (40, 41). We saw similar baseline-corrected normalized fluorescence intensity changes induced by single AP-stimulation before and 1 min after incubation with 20 µM octopamine (*SI Appendix*, Fig. S1*D*, see the same panel for control condition without octopamine). Prolonged stimulation (20 APs @ 20 Hz) also elicited similar signals (*SI Appendix*, Fig. S1*E*). Thus, we found no evidence that AP-induced Ca^2+^ levels were altered by octopamine.

Increasing the number of occupied neurotransmitter release sites at any given AZ also potentiates neurotransmitter release. Unc13 is a limiting component of these sites and recruited to the AZ by the scaffolding protein BRP (42–44). We therefore tested whether acute octopamine treatment affected immunofluorescent signals for either protein at NMJs. Quantitative confocal imaging revealed higher BRP and Unc13A NMJ signals in animals treated with octopamine for 1 min prior to fixation compared to ones receiving a mock (DMSO) treatment (Fig. 1*G*). Longer octopamine exposure did not increase signals further (30 min, *SI Appendix*, Fig. S3). Thus, octopamine rapidly alters both the physiological output and the structure of the synapse.

### Rapid Nanoscale Enrichment of Unc13 with Octopamine Treatment.

**Fig. 2.**
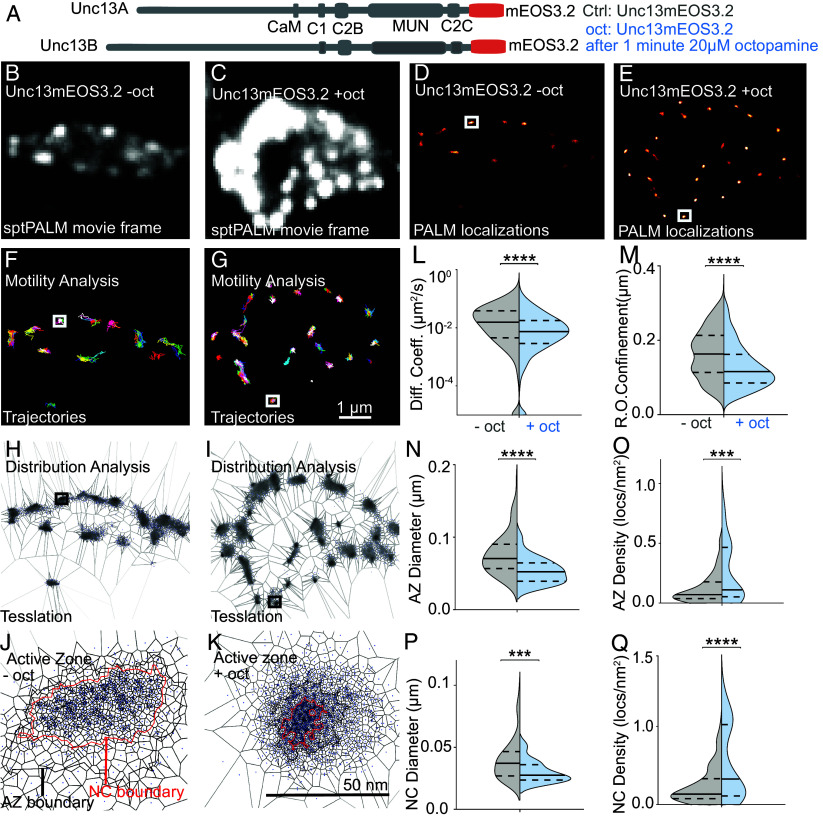
Live in vivo imaging of endogenously tagged Unc13 molecules reveals their immobilization and compaction upon acute octopamine treatment. (*A*) Scheme of Unc13A and Unc13B protein illustrating the mEOS3.2 tagging. (*B* and *C*) Cumulative intensity Images from live sptPALM recordings of Unc13mEOS3.2 show boutons before (*B*, −Oct) and after (*C*, +Oct) 1-min octopamine (20 μM) incubation. (*D* and *E*) PALM localization maps of the same boutons before (*D*, -Oct) and after (*E*, +Oct) octopamine treatment. (*F* and *G*) trajectory maps before (*F*, -Oct) and after (*G*, +Oct) treatment. (*H*–*K*) Tessellation analysis representation of a bouton (*H* and *I*) and individual AZs depicting AZ cluster (black) and nanocluster (NC, red) boundaries. (*L* and *M*) Quantification of diffusion coefficients (*L*) and radii of confinement (*M*) before (−Oct, gray) and after (+Oct, blue) treatment. (*N* and *O*) Quantification of Unc13mEOS3.2 localizations within AZ cluster boundaries (black border in *J* and *K*) for diameter (*N*) and localization density (*O*) before (−Oct, gray) and after (+Oct, blue) treatment. (*P* and *Q*) same as (*N* and *O*) for nanocluster boundaries (red border in *J* and *K*). Number of NMJs (n), animals (N), AZs (X), and individual trajectories (Y) investigated: n/N/X/Y(−Oct) = *7*/7/106/2051; n/N X/Y (+Oct) = 7/7/86/2505; Data depict mean values ± SEM. Statistical significance is denoted as asterisks: **P < 0.01, ***P < 0.001, and ****P < 0.0001. Data distribution was statistically tested with a Kolmogorov–Smirnov test. For details, see *SI Appendix*, Table S6. AU, arbitrary units. [Scale bars, 1 μm (*B*–*G*) and 50 nm (*H* and *I*).]

We wanted to test whether the dynamics or nanoscopic structure of Unc13 changed on this rapid timescale and therefore performed live single molecule imaging of endogenously labeled Unc13 (fused to an mEOS3.2 tag). Tagging did not affect basal synaptic properties, structure, or function (*SI Appendix*, Fig. S4). To capture rapid nanoscale adaptation of Unc13 distribution during octopamine application, we performed live sptPALM Unc13mEOS3.2 imaging at Ib type muscle 4 NMJs before and immediately after 20 μM octopamine application in the same animal at an acquisition rate of 20 Hz (Fig. 2 and *SI Appendix*, Table S2, further details described in the *Material and Methods* section).

Notably, octopamine treatment reduced Unc13 motility, evident in a lower median diffusion coefficient (Fig. 2*L*). Also, the mean radius of confinement of Unc13 trajectories significantly decreased following octopamine treatment (Fig. 2*M*). We further used an SR tessellation analysis to calculate the nanoscale distribution of Unc13 from PALM localization data by calculating the diameters and densities occupied by Unc13 localizations. We investigated “AZ clusters” and smaller “Nanoclusters” (9) (see *Material and Methods* for criteria). Control experiments established that none of these parameters were affected by the repeated imaging protocol itself (*SI Appendix*, Fig. S5). Following octopamine treatment, the diameters of AZ- and nanoclusters decreased, while the signal density within those increased (Fig. 2 *N*–*Q*). The number of Unc13mEOS molecules per AZ estimated in bleach curve analysis did not change (*SI Appendix*, Fig. S6). Longer term (10 min) octopamine treatment resulted in similar changes, as did a 1 min of phorbol ester Phorbol-12-Myristat-13-Acetat (PMA) treatment which also potentiates release at the NMJ (45, 46) (*SI Appendix*, Fig. S7). Thus, the immobilization and nanoscale compaction of Unc13 correlates with synaptic potentiation induced by different treatments.

### Octopamine-Induced Potentiation Depends on Presynaptic Unc13A.

**Fig. 3.**
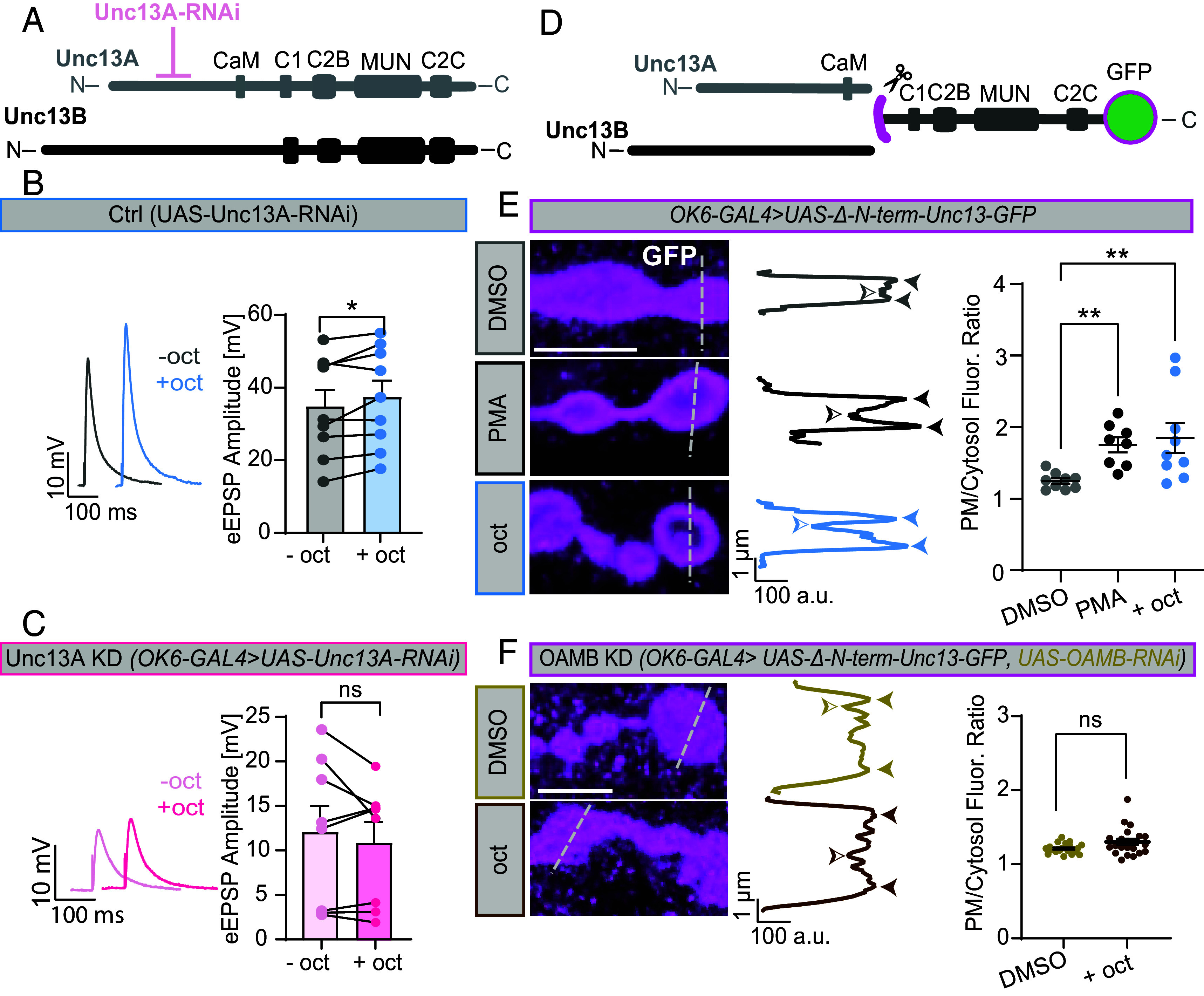
Octopamine-induced synaptic potentiation requires Unc13A, and a C-terminal Unc13 fragment is recruited to the plasma membrane by octopamine dependent on the OAMB receptor. (*A*) Scheme of Unc13A and -B isoforms and the RNAi approach for Unc13A knockdown. (*B*) Representative eEPSP responses and cell-wise comparison of eEPSP amplitudes in control (Ctrl) synapses before (−oct, gray) and after octopamine incubation (+oct, blue). (*C*) same as (*B*) following Unc13A knockdown (Unc13A KD, −oct, light pink; +oct, pink). (*D*) Scheme of truncated Unc13 fragment with C-terminally fused GFP. (*E*, *Left*) Representative confocal NMJ images of GFP signal at muscle 4 NMJs (segment A3–5) presynaptically expressing the labeled Unc13 fragment. Larvae were either incubated with DMSO alone (DMSO, 1 min, gray), with PMA (2 µM, 10 min, black), or octopamine (+oct, 20 µM, 1 min, blue). GFP intensity profiles were collected at dashed lines. (*Middle*) Line profiles from respective images representing Unc13-GFP fluorescent intensities. The ratio between highest and lowest intensities (filled/open arrows) was quantified (*Material and Methods*). (*Right*) Quantification of intensity ratios across NMJs. (*F*) Same as *E*, but following motoneuron OAMB knockdown treated either with DMSO alone (DMSO, 1 min, yellow) or octopamine (20 µM, 1 min, brown). Number of cells (n) and animals (N) investigated: n/N[Ctrl (UAS-Unc13A-RNAi), B] = 9/9; n/N(Unc13A KD, C) = 8/8; n/N(DMSO, E) = 9/3; n/N(PMA, E) = 8/3; n/N(oct, E) = 9/3; n/N(DMSO OAMB KD, F) = 23/8; n/N(oct OAMB KD, F) = 22/8. For exact genotypes, see *Material and Methods*. Data depict mean values ± SEM. Statistical analysis with unpaired, Mann–Whitney *U* test (*E* and *F*) or with paired parametric *t* tests (*B* and *C*). For details, see *SI Appendix*, Table S11. n.s., *P* > 0.05; *P 0.05; ***P 0.001. [Scale bar in (*F* and *G*): 5 µm.]

The principal splice isoforms of Unc13, Unc13A, and -B are both present at larval NMJs (Fig. 3*A*), and both isoforms are tagged in the experiments above. However, neurotransmitter release at this synapse predominantly depends on Unc13A (44). To explore whether Unc13A plays a direct role in this rapid potentiation, we tested whether potentiation was disrupted upon Unc13A knockdown (KD) in motoneurons (Unc13A KD, *OK6-Gal4>UAS-Unc13A-RNAi*) (47). Compared to control animals (*UAS-Unc13A-RNAi* without OK6Gal4), presynaptic Unc13A KD increased mEPSP amplitudes, and decreased eEPSP amplitudes (*SI Appendix*, Fig. S8*B*) in qualitative agreement to the Unc13A null mutation (44). While neurotransmitter release was potentiated at NMJs from control animals upon 1-min 20 µM octopamine treatment (Fig. 3*B*) (similarly as in wildtype animals, Fig. 1*A*), no potentiation was seen upon presynaptic Unc13A KD (Fig. 3*C*). This points to a direct role of Unc13A in mediating the fast neurotransmitter potentiation by octopamine. This is consistent with a recent study that also identified Unc13A, and not Unc13B, as a target for octopamine-induced potentiation using optical quantal imaging (34).

### Octopamine Signaling Recruits a Delocalized Unc13 C-Terminal Fragment to Synaptic Plasma Membranes.

Because Unc13A levels correlate with synaptic strength and BRP recruits Unc13A via interactions with the Unc13A N terminus (42–44), we wondered whether potentiation was secondary to increased BRP levels, or whether octopamine engaged Unc13 directly. We uncoupled its interaction with BRP by deleting the Unc13 N terminus in a GFP-tagged C-terminal fragment (Fig. 3*D*) overexpressed in motoneurons (using the *OK6-Gal4* driver) to investigate whether 1-min application of octopamine resulted in any detectable change in protein localization. This construct is delocalized at rest (Fig. 3 *E* and *F*) (43) similar to a fragment of the mammalian ortholog Munc13 which translocates to the plasma membrane in cells treated with phorbol esters (48). This was also the case for our construct at the NMJ following incubation with PMA (10 min, 2 µM) (Fig. 3*E*). To quantitatively compare this behavior, we calculated the fluorescence intensity ratios between bouton edges (plasma membrane) and cytoplasm (bouton center). This revealed that 1-min exposure to 20 µM octopamine similarly redistributed the Unc13 C-terminal fragment to the plasma membrane as PMA (Fig. 3*E*). To check whether this signaling depended on the OAMB receptor, we repeated the experiment following its RNAi-mediated knockdown in motoneurons. This blocked the octopamine-induced translocation of the Unc13 C-terminal fragment seen in control animals (Fig. 3*F* and *SI Appendix*, Fig. S8*G*). Thus, octopamine triggers the plasma membrane recruitment of the C-terminal portion of Unc13 in an OAMB receptor-dependent manner.

### Mutations in the Unc13 Lipid Binding C2B and C1 Domains Enhance Neurotransmitter Release, Reduce Synaptic Unc13A Signals, and Attenuate Octopamine-Induced Potentiation.

**Fig. 4.**
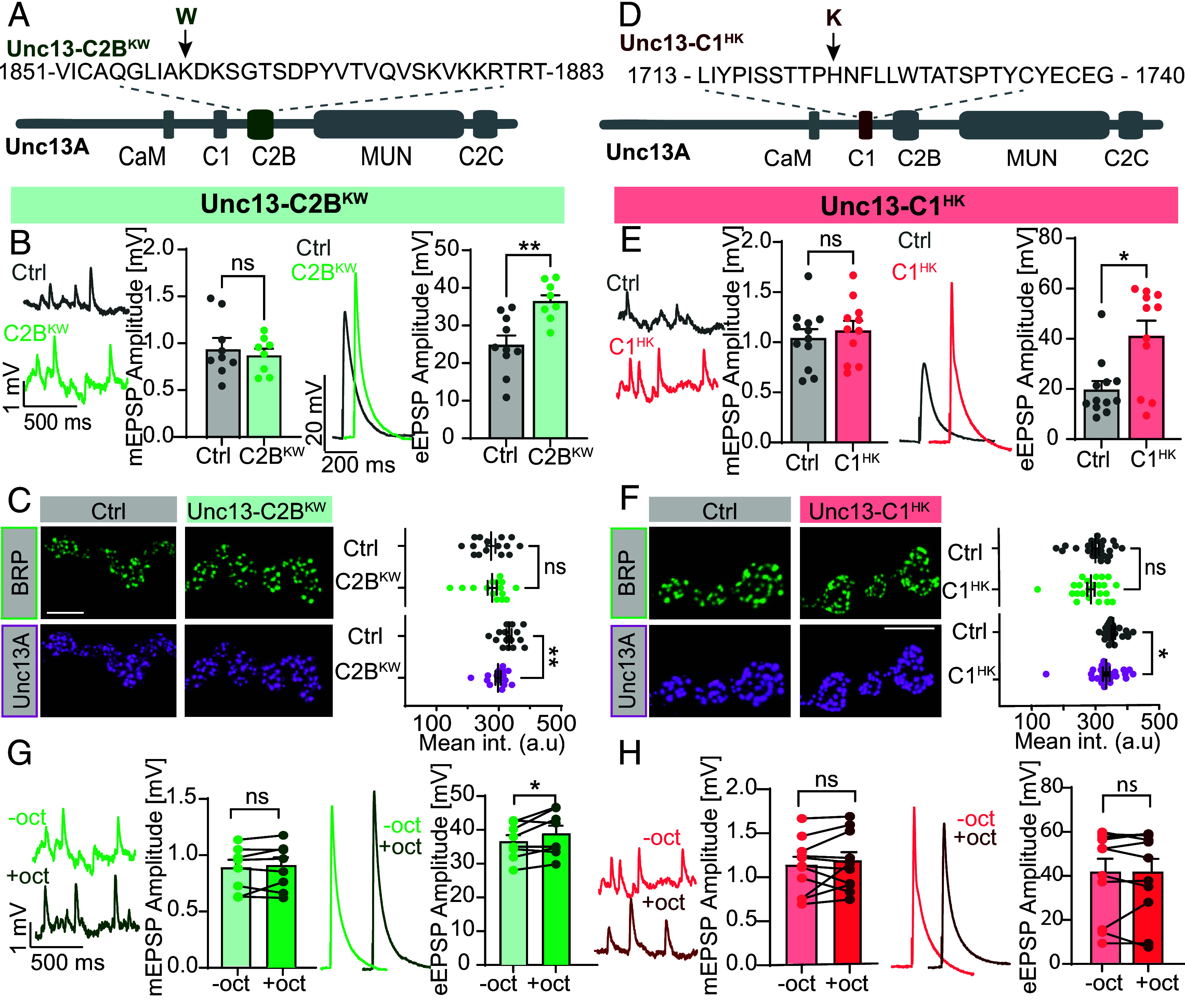
Enhanced neurotransmitter release, reduced Unc13A signals, and diminished octopamine-induced potentiation in Unc13-C1^HK^ and Unc13-C2B^KW^ mutants. (*A*) Schematic representation of Unc13-C2B^KW^ (K1862W in Unc13A) mutant. (*B*, *Left*) representative traces of spontaneous mEPSPs from wildtype (Ctrl, w1118, gray) and Unc13-C2B^KW^ (light green) mutant synapses with cell-wise quantification of mEPSP amplitudes. (*Right*) Representative AP-evoked eEPSP responses from wildtype (Ctrl, w1118, gray) and Unc13-C2B^KW^ (light green)mutant synapses with cell-wise quantification of eEPSP amplitudes. (*C*) Representative confocal images (*Left*) of muscle 4 NMJs from wildtype (Ctrl, w1118, gray) and Unc13-C2B^KW^ (light green label) stained with antibodies against BRP (green, *Top*) and the N-terminal region of Unc13A (magenta, *Bottom*, see *Material and Methods*). (*Right*) Quantification of mean BRP (*Top*) and Unc13A (*Bottom*) intensities. (*D*–*F*) same as (*A*–*C*) for the Unc13-C1HK (H1723K in Unc13A) mutant (traces and labels in light red). (*G* and *H*, *Left*) Representative traces of spontaneous mEPSPs and cell-wise quantification of mEPSP amplitudes before (−oct, light green/red) and 1-min after 20 µM octopamine (+oct, dark green/red) at Unc13-C2B^KW^ (*G*, green) or Unc13-C1^HK^ (*H*, red) mutant synapses. (*Right*) Representative AP-evoked eEPSP responses before octopamine (−oct, light green/light red) and after 1-min incubation with 20 µM octopamine (+oct, dark green/dark red) and quantification of eEPSP amplitudes at Unc13-C2B^KW^ (*G*, green) or Unc13-C1^HK^ (*H*, red) mutant synapses. (*B*, *E*, *G*, and *H*) Current clamp analysis (muscle 6 NMJs, 0.4 mM extracellular Ca^2+^). Number of cells (n) and animals (N) investigated: n/N: n/N(*Ctrl for Unc13-C2B^KW^, B*) *=* 10/10 (eEPSP) and 9/9 (mEPSP), n/N(*Unc13-C2B^KW^*, *B* and *G*) = 8/8(eEPSP) and 8/8 (mEPSP), n/N(*Ctrl for Unc13-C ^HK^, E*) = 12/12 (eEPSP) and 12/12 (mEPSP), n/N(*Unc13-C1^HK^*, *E* and *H*) = 11/11, n/N(Ctrl, *C*) = 15/5; n/N(Unc13-C2B^KW^, *C*) = 15/5, n/N(Ctrl, *F*) = 24/8, n/N(Unc13-C1^HK^, *F*) = 23/8. For exact genotypes, see *Material and Methods*. Data depict mean values ± SEM. Statistical analysis with unpaired, Mann–Whitney *U* test (*B*, *C*, *E*, and *F*) or with paired parametric *t* tests (*G* and *H*). For details, see *SI Appendix*, Table S13. n.s., *P* > 0.05; *P 0.05; **P 0.01. [Scale bar in (*C* and *F*): 5 µm.]

We wanted to investigate whether Unc13’s C1 and C2B domains, which bind to the two signaling lipids of the Gαq pathway (DAG and PI(4, 5)P_2_) contribute to octopamine-induced potentiation. Both domains are needed for membrane binding and were shown to exert autoinhibitory functions (49–51). However, the cellular signaling pathways that modulate synaptic function via these domains have not been identified. We generated mutant flies by CRISPR Cas9 gene editing of specific amino acids in the two domains. As both domains are present in either Unc13 isoform, this approach targeted both Unc13A and -B. In the C2B domain mutant, a central lysine was mutated to a tryptophane (Unc13-C2B^KW^; K1862W in Unc13A) (Fig. 4*A*) which increases the PI(4, 5)P_2_-binding affinity and enhances synaptic transmission in the mammalian ortholog (51). The mutation in the C1 domain changed a central histidine to a lysine (Unc13-C1^HK^; H1723K in Unc13A) (Fig. 4*D*) which enhances synaptic transmission in central mouse neurons (14, 15, 52). We first confirmed that these mutations also enhanced neurotransmitter release in flies. Current clamp recordings indeed revealed increased eEPSPs for both mutants when compared to wildtype animals (Fig. 4 *B* and *E*, *Right*). At the same time, no detectable changes of mEPSP amplitudes or frequencies were seen (Fig. 4 *B* and *E*, *Left* and *SI Appendix*, Fig. S9). Confocal microscopy following immunostaining against BRP and Unc13A revealed lower Unc13A signals in both the Unc13-C2B^KW^ and Unc13-C1^HK^ mutants compared to control animals (wildtype, w1118), whereas BRP signals were similar (Fig. 4 *C* and *F*). We next investigated the reactivity of the Unc13-C2B^KW^ and Unc13-C1^HK^ mutants to 1-min 20 µM octopamine incubation. While octopamine still potentiated neurotransmitter release in animals expressing the Unc13-C2B^KW^ mutation (Fig. 4*G*), potentiation was lost in Unc13-C1^HK^ mutants (Fig. 4*H*).

A concern with the elevated baseline eEPSP responses in Unc13-C1HK mutants is that octopamine-induced potentiation may be masked by response saturation. Indeed, in control NMJs, the strongest potentiation typically occurs when baseline responses are low (Fig. 1*B* and *SI Appendix*, Fig. S10*C*). Two-electrode voltage clamp (TEVC) experiments further support this: They revealed robust potentiation at low extracellular Ca^2+^ (0.4 mM), but none at higher Ca^2+^ (1.5 mM) (*SI Appendix*, Fig. S11*B*). We interpret this as a ceiling effect, where elevated baseline release limits the dynamic range for further enhancement—a phenomenon also observed with phorbol ester–induced potentiation (45). To test whether such saturation alone accounts for the lack of potentiation in Unc13-C1HK mutants, we recorded eEPSPs at 0.1 mM Ca^2+^ and eEPSCs at 0.4 mM Ca^2+^—both well below saturation levels in wildtype synapses (*SI Appendix*, Figs. S10 *A* and *B* and S11 *A* and *C*). However, octopamine-induced potentiation remained absent in the mutants. This suggests that while the magnitude of potentiation depends on the baseline state, the C1 domain mutation specifically impairs the ability to potentiate—potentially by blocking activation of the pathway or by mimicking a prepotentiated state.

**Fig. 5.**
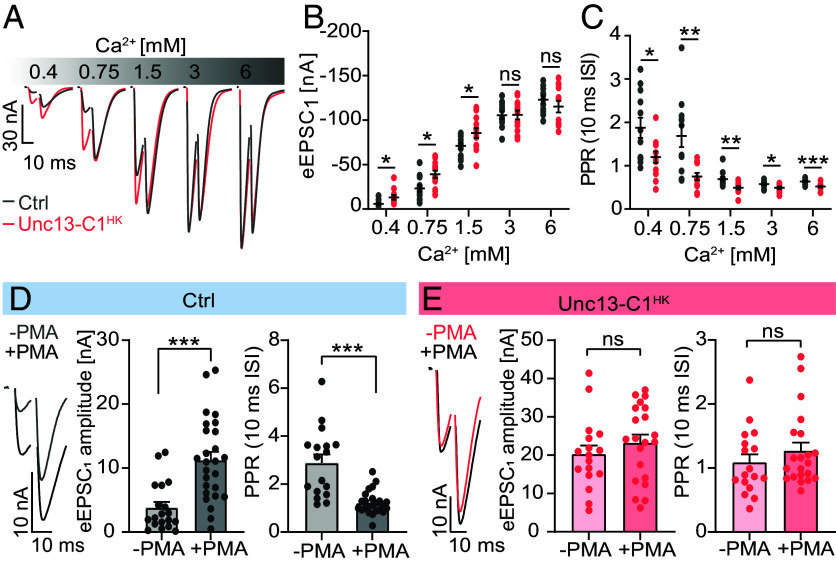
The Unc13-C1^HK^ mutation enhances immediate neurotransmitter release at physiological Ca^2+^ concentrations and below, abolishes short-term facilitation, and blocks phorbol ester–induced potentiation (*A*) Representative example TEVC traces displaying AP-evoked responses to paired AP stimulation (10 ms interstimulus interval) from a single wildtype (Ctrl, w1118, gray) and Unc13-C1^HK^ (red) NMJs, acquired at progressively increasing extracellular Ca^2+^ concentrations ([Ca^2+^]_E_). Quantification of first (*B*) eEPSC_1_ amplitudes and (*C*) PPRs in Ctrl (gray) and Unc13-C1^HK^ (red) conditions. (*D* and *E*) Analysis of PMA-potentiation in Ctrl (*D*, w1118) and Unc13-C1^HK^ (*E*) animals at 0.4 mM extracellular Ca^2+^ concentration. (*Left*) Representative traces of AP-evoked paired-pulse responses recorded after a 10-min preincubation with either DMSO (−PMA) or 2 μM PMA in DMSO (+PMA). Middle: quantification of eEPSC_1_ amplitudes. (*Right*) quantification of PPR (PPR = eEPSC_2_/eEPSC_1_) (10 ms interstimulus interval [ISI]). Number of cells (n) and animals (N) investigated: n/N: n/N(Ctrl, *B* and *C*) = 12/12, n/N(Unc13-C1^HK^, *E*) = 12/12, n/N(Ctrl -PMA, *D*) = 19/9, n/N(Ctrl +PMA, *D*) = 26/11, n/N(Unc13-C1^HK^ −PMA, *E*) = 17/7, n/N(Unc13-C1^HK^ +PMA, *E*) = 21/7. For exact genotypes, see *Material and Methods*. Data depict mean values ± SEM. Statistical analysis with Mann–Whitney *U* test (en rule) (*B*–*E*). For details, see *SI Appendix*, Table S17. n.s., *P* > 0.05; *P 0.05; **P 0.01; ***P 0.001.

We next analyzed whether the Unc13-C1^HK^ mutation affected short-term plasticity (STP) by measuring eEPSCs in response to paired AP-stimuli (10 ms apart) at successively increased Ca^2+^ concentrations in the external medium (Fig. 5*A*). Comparison of the amplitude of the first evoked EPSC (eEPSC_1_) revealed larger responses in Unc13-C1^HK^ mutants at physiological (1.5 mM) external Ca^2+^ concentrations and below (Fig. 5*B*). The mutation also affected STP by lower paired-pulse ratios (PPRs) of the eEPSC amplitudes successively induced by repetitive stimulation (PPR = eEPSC_2_/eEPSC_1_) at all investigated Ca^2+^ concentrations (Fig. 5*C*). We confirmed that like in the case of the mammalian ortholog (14) phorbol ester application (PMA, 2 μM for 10 min)—which profoundly increased eEPSC_1_ amplitudes and decreased PPRs in wildtype control NMJs—had no effect in Unc13-C1^HK^ mutants (Fig. 5 *D* and *E*) (45).

Octopamine is secreted from type II boutons during starvation states which enhances locomotion speed via the Octβ2R receptor (16). We wondered whether the pathway described here also contributed to this behavioral adaptation and therefore tested whether this was affected in Unc13-C1^HK^ mutants. Comparison of fed third-instar larvae revealed slower crawling of Unc13-C1^HK^ mutants (*SI Appendix*, Fig. S12*A*). However, starvation for 2 h robustly increased locomotor speeds in both mutant and wildtype larvae (*SI Appendix*, Fig. S12*B*), suggesting that this behavioral adaptation can be achieved without regulation of the Unc13-C1 domain. Moreover, OAMB receptor knockdown in type I motoneurons (*OK6-Gal4>UAS-OAMB-RNAi*) neither affected crawling speeds in fed animals (*SI Appendix*, Fig. S12*C*) nor did it affect the starvation-induced locomotion (*SI Appendix*, Fig. S12*D*). These results are consistent with a separate pathway involving Octβ2R/Octβ1R and cAMP for this behavioral adaptation (16, 17).

### Mutation of the Unc13 C1 Domain Blocks Octopamine-Induced Nanoscale Compaction and Reduces Unc13 Copy Numbers.

**Fig. 6.**
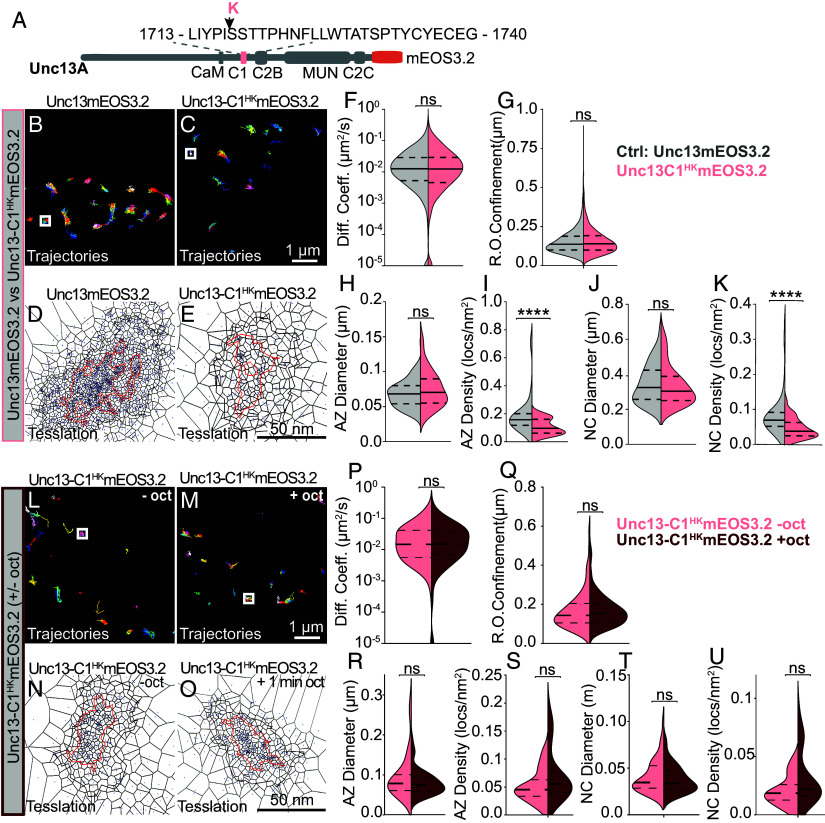
Live in vivo imaging of Unc13-C1^HK^mEOS reveals loss of octopamine-induced Unc13 compaction at AZs. (*A*) Scheme of Unc13A protein showing its regulatory domains and the C-terminal mOES3.2 tag of endogenous Unc13. The arrow indicates the amino acid change in the C1 domain in the Unc13-C1^HK^ mutant protein. (*B*–*K*) Genotype comparison between Unc13mEOS3.2 and Unc13-C1^HK^mEOS3.2. (*L*–*U*) Comparison of Unc13-C1^HK^mEOS3.2 before (−oct) and after (+oct) a 1-min octopamine. (*B*, *C*, *L*, and *M*) trajectory maps. (*D*, *E*, *N*, and *O*) Tessellation representation of AZ cluster (black) and nanocluster (NC, red). (*F*, *G*, *P*, and *Q*) Quantification of diffusion coefficients and radii of confinement. Tessellation analysis for diameters and localization densities within AZ (*H*, *I*, *R*, and *S*) and NC cluster (*J*, *K*, *T*, and *U*) boundaries. Number of NMJs (n), animals (N), number of AZs (X), and the number of individual trajectories (Y) investigated: experiment 1: n/N/X/Y (Unc13-C1 ^HK^mutant-Oct) = 10/6/108/5832; n/N/X/Y (Unc13-C1 ^HK^ mutant +Oct) = 10/6/111/2306 and experiment 2: n/N/X/Y (Unc13-C1 ^HK^mutant-Oct) = 6/6/45/264; n/N/X/Y (Unc13-C1^HK^mutant +Oct) = 7/7/52/183. Statistical significance is denoted as asterisks: **P < 0.01, ***P < 0.001, and ****P < 0.0001. Data distribution was statistically tested with a Kolmogorov–Smirnov test. For details, see *SI Appendix*, Table S19. AU, arbitrary units. [Scale bars, 1 μm (*B* and *C*) and 50 nm (*D* and *E*).]

We also generated an endogenously tagged Unc13-C1^HK^-mEOS3.2 mutant and compared the mobility and nanoscale localization of this mutated version to nonmutated Unc13-mEOS3.2. Diffusion coefficients and AZ-/Nanocluster sizes were similar (Fig. 6 *A*–*K*), but the protein density was reduced for the Unc13-C1^HK^, consistent with the lower signals seen in confocal analysis (Fig. 4*F*). This was further supported by a significant reduction in the number of Unc13-C1^HK^-mEOS3.2 proteins detected by stepwise photobleaching (*SI Appendix*, Fig. S13. Upon octopamine treatment, the Unc13-C1^HK^-mEOS3.2 mutant neither underwent compaction nor motility changes (Fig. 6 *L*–*U*). This aligns with the blocked eEPSP potentiation in this condition (Fig. 4*H*) though the absence of compaction despite elevated release at rest suggests that additional factors or adaptive mechanisms may influence Unc13’s nanoscale organization in this mutant (*Discussion*).

### Octopaminergic Modulation Intersects with Homeostatic Plasticity Mechanisms.

**Fig. 7.**
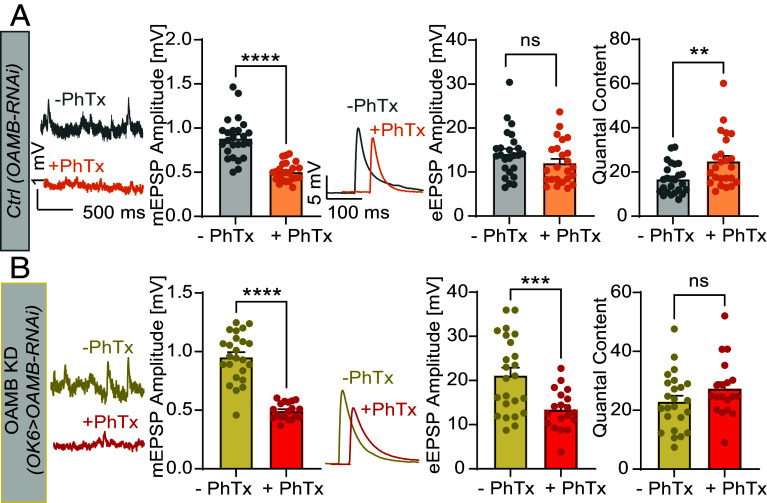
PHP depends on the expression of the OAMB receptor in motoneurons. (*A* and *B*) Analysis of PhTx effects in current clamp recordings (muscle 6 NMJs, 0.2 mM extracellular Ca^2+^) of spontaneous and AP-evoked synaptic activity in control animals (UAS-OAMB-RNAi, *A*) and OAMB KD animals (OK6-GAL4>UAS-OAMB-RNAi, *B*). (*Left*) Representative example traces of mEPSPs and cellwise quantification of mEPSP amplitudes from control (UAS-OAMB-RNAi, *A*) or Oamb KD animals (OK6-GAL4>UAS-OAMB-RNAi, *B*) after 10 min incubation either with 20 μM PhTx (+PhTx) or without (−PhTx). Middle: Representative eEPSP responses (average of five repetitions in one cell) from control synapses −PhTx (gray/yellow) or +PhTx (orange, red) and quantification of eEPSP amplitudes (−PhTx, gray/yellow; +PhTx, orange/red), and the quantal content (eEPSP/mEPSP) with and without 10-min 20 µM PhTx treatment. Number of cells (n) and animals (N) investigated: n/N(UAS-OAMB-RNAi −PhTX) = 25/9, n/N(UAS-OAMB-RNAi +PhTX) = 24/8, n/N(OK6-GAL4>UAS-OAMB-RNAi −PhTx) = 24/8; n/N(OK6-GAL4>UAS-OAMB-RNAi −PhTx) = 19/7 For exact genotypes, see *Material and Methods*. Data depict mean values ± SEM. Statistical analysis with unpaired, *t* test. For details, see *SI Appendix*, Table S22. n.s., *P* > 0.05; *P 0.05; **P 0.01.

To explore whether octopaminergic signaling shares features or molecular components with other forms of synaptic plasticity, we examined its relationship to PHP at the NMJ. Recently, single molecule imaging of Unc13 fixed and immunolabeled molecules revealed its compaction at the NMJ 10 min after treatment with the PHP-inducing glutamate receptor antagonist Philanthotoxin (53). In line with this, PhTx-exposure (20 μM, 10 min) reduced Unc13A-mEOS3.2 motility and increased its compaction in our live imaging approach (*SI Appendix*, Fig. S14), closely resembling the nanoscale changes observed after octopamine or PMA treatment (Fig. 2 and *SI Appendix*, Figs. S6 and S7). Similarly, PhTx promoted translocation of the C-terminal Unc13-GFP fragment to the presynaptic membrane (*SI Appendix*, Fig. S8*H*), further supporting convergence in the structural mechanisms of potentiation. Finally, PhTx-induced PHP (seen in an elevated quantal content to compensate reduced mEPSP amplitudes) was blocked in animals with motoneuron-specific knockdown of OAMB receptors (*OK6-Gal4>UAS-OAMB-RNAi*) but present in control animals (Fig. 7). Together, these findings suggest that modulation of nanoscale Unc13 localization is a shared feature of multiple potentiation pathways and suggest that components of the monoaminergic signaling pathway also contribute to homeostatic plasticity.

## Discussion

We here set out to investigate mechanisms of fast neurotransmitter release potentiation. The monoamine octopamine increases neurotransmitter release at the *Drosophila* third-instar larval NMJ within 1 min. Potentiation depended on PLC activity, and while no significant alterations of AP-induced Ca^2+^ levels were seen, the confocal signals of BRP and Unc13 increased with 1-min octopamine treatment. A dependence on Unc13 for this fast potentiation was corroborated genetically and identified a pivotal role of the DAG binding Unc13 C1 domain. A point mutation in the domain not only blocked the potentiation by octopamine and DAG analogs but also increased baseline neurotransmitter release, lowered PPR values, and reduced synaptic Unc13 signals. Live, single molecule microscopy revealed a rapid rearrangement of the Unc13 on the nanoscale coincident with potentiation by octopamine, PMA, and PhTx (Figs. 2 and 6 and *SI Appendix*, Figs. S5–S7, S13, and S14). A connection between PHP and this pathway was implied by the shared dependence on presynaptic OAMB receptors.

We here exclusively focused on type I larval NMJs at muscles 4 and 6. These muscles are not contacted by the peptidergic type II NMJs known to release octopamine (16, 31, 54). The *Drosophila* genome encodes five different receptors, three beta-adrenergic-like Receptors (OctβRs), and two alpha-adrenergic-like receptors, OAMB and Octα2R with widespread distribution throughout the nervous system (55). At larval NMJs, acute octopamine application elevates cAMP levels (of muscle 12), triggers neuropeptide release, and enhances glutamate release (16, 32, 33, 35). Type IB motoneurons express Octβ1R, Octβ2R, and OAMB at similar levels and OAMB expression is needed for presynaptic potentiation (Fig. 1*C*) (34, 38). While our experiments rely on bath application of octopamine, the rapid onset of potentiation and nanoscopic remodeling observed suggests that under physiological conditions, brief, spatially confined release events may be sufficient to engage this pathway. However, the precise source, timing, and synaptic specificity of endogenous octopamine signaling at the larval NMJ remains to be established. Our attempts to visualize discrete octopaminergic input sites by immunostaining were inconclusive, potentially due to low abundance or widespread diffusion. Future studies using recently developed octopamine sensors (56) may help resolve where and in which physiological contexts octopamine is released in vivo to engage this pathway. Notably, a recent study at the *Drosophila* NMJ revealed that cAMP signaling triggers Unc13A reorganization and synaptic potentiation via Rab3 (57), highlighting a parallel structural mechanism for presynaptic plasticity through a distinct GPCR cascade. In central circuits, octopamine has similarly been shown to regulate active zone BRP architecture via Octβ2R in a compartment-specific manner (58), supporting a broader role for octopaminergic modulation in tuning presynaptic structure and output across neuronal systems.

Although fast octopamine-induced potentiation was fully blocked by pharmacological PLC inhibition (Fig. 1*D*), RNAi expression in motoneurons merely attenuated it (Fig. 1*E* and *SI Appendix*, Fig. S2 *G* and *H*), possibly due to incomplete knockdown. In support of the direct role of PLC/DAG signaling lipid in this fast neuromodulation, the mutation of the DAG-binding Unc13 C1 domain fully blocked potentiation (Fig. 4*H* and *SI Appendix*, Fig. S9*B*). We cannot exclude the relevant role of additional downstream PLC targets and indeed expect that these will be needed for long-term potentiation. Indeed, IP_3_ functions in long-term PHP (59) and PKC has several synaptic phosphorylation targets, including (M)Unc18, Synaptotagmin-1, and SNAP25 whose phosphorylation affects neurotransmitter release (24, 25, 60). Future work will establish which signaling pathways and messengers mediate distinct phases of potentiation.

PMA strongly and rapidly potentiates neurotransmitter release (Fig. 5) and we here show that it similarly reduces Unc13 motility and enhances its nanoscopic concentration as octopamine and PhTx (Fig. 2 and *SI Appendix*, Fig. S14). The Unc13-C1^HK^ mutation blocked PMA and octopamine induced potentiation at the NMJ, suggesting that the mutation either blocks or preactivates the pathway (Fig. 5 and *SI Appendix*, Fig. S11). Although this mutation functionally mimics a potentiated state, it neither reduced Unc13 motility nor induced the compaction observed upon acute potentiation of wildtype synapses (Figs. 2 and 6). This suggests that Unc13 compaction may be a hallmark of early-phase potentiation, while chronic activation—as imposed by the mutation—triggers compensatory processes that restore a more normal Unc13 nanoscale distribution and dynamics. However, a comparison between mutant and wildtype synapses revealed a severe reduction in the number of Unc13 molecules assessed by bleach curve analysis (*SI Appendix*, Figs. S6 and S13). Notably, lower confocal signals of antibody-labeled Unc13 were observed for mutants of the Ca^2+^ calmodulin binding domain, the C2B and the C1 domain which all had increased baseline responses and lower capacity to be potentiated by PMA and/or octopamine [Figs. 4 *E* and *H* and 5*E*, (45)]. While it is typically observed that AZ protein levels increase with potentiation, this indicates the opposite: Lower protein levels in cases where baseline transmission is enhanced, opening up the interesting possibility of bidirectional homeostasis. Consistent with this, the overall effects of the mutations were low at physiological external Ca^2+^ concentrations (Fig. 5 and *SI Appendix*, Fig. S10*B*) (45), which could illustrate an attempt to normalize synaptic communication in this range (i.e., 1.5 mM Ca^2+^). These homeostatic changes, including the reduction in Unc13 abundance, may mask the full gain-of-function effect. Our findings thus highlight how the temporal dynamics of Unc13 regulation—acute versus chronic—can shape distinct structural and functional outcomes.

A recent study by Bakshinska et al. (34) independently examined the Unc13 C1 domain using an RNAi/rescue approach and reported related mechanistic conclusions on the role of the OAMB receptor, PLC and the Unc13-C1 domain. While they did not observe elevated baseline release, this may reflect differences in expression levels or relative proportions of endogenous and mutant protein. In contrast, our CRISPR-based point mutation at the endogenous locus consistently produces a gain-of-function phenotype at low extracellular Ca^2+^ concentrations (0.1 to 0.4 mM), while differences diminish at 1.5 mM Ca^2+^, matching their experimental conditions. We view the convergence of results across these distinct experimental strategies as strong, complementary evidence for the role of the C1 domain in monoaminergic synaptic potentiation.

Besides disrupting octopamine-induced potentiation, the Unc13-C1^HK^ mutation also caused short term synaptic depression. A marked decrease in locomotor speed was seen (*SI Appendix*, Fig. S12 *A* and *B*). Loss of octopaminergic input also decreases larval crawling speeds (61), but this is unlikely to rely on OAMB-dependent signaling at the level of type I motoneurons since we observed no effect of its knockdown (*SI Appendix*, Fig. S12 *C* and *D*). Given the marked change in short-term plasticity, we predict that this mutation likely affects the temporal pattern of motoneural activation, which is consistent with the observed disruption of coordinated movement in *C. elegans* and human patients with (M)Unc13 mutations (49, 62). Future research investigating the effect of this mutation on the central pattern generator for locomotion may thus provide valuable insight into the relevance of Unc13 modulation for coordinated movements.

Previously reported synaptic structural changes typically occur over longer timescales—for example, octopamine-induced growth over days (16), or long-term potentiation accumulating Munc13 and shortening coupling distances to Ca^2+^ channels in the hippocampus (8, 63). Alignments between Munc13–postsynaptic receptor undergoes changes within 25 min (64), and similarly, live single-molecule imaging at the fly NMJ revealed nanoscopic compaction of Ca^2+^ channels and BRP during sustained PHP within 20 to 30 min (9). Several studies report PHP-related structural changes within 10 min (5, 10, 65).

Here, we observed increased Unc13A confocal signals after just 1-min octopamine treatment (Fig. 1*G*), with no further increase at later times (*SI Appendix*, Fig. S3). This was corroborated by sptPALM which demonstrated instant slowing of Unc13 motility and its signal compaction within AZs, without a change in relative molecule numbers (Fig. 2 and *SI Appendix*, Fig. S6). This may reflect an early potentiation phase involving AZ compaction—as described after 10-min PHP (53)—later followed by protein accumulation, as seen for BRP and Cac at 20 min (9). Supporting this, recent work shows chronic and acute PHP remodel distinct inputs (MN-Ib versus MN-Is) via different mechanisms, including Unc13A accumulation and AZ compaction (66).

Our data suggest mechanistic convergence between octopamine-induced potentiation and PHP. PhTx, like octopamine and PMA, reduced Unc13 motility, drove compaction, and translocated the Unc13 C-terminal fragment to the presynaptic membrane (Figs. 2, 3*E*, and 6 and *SI Appendix*, Figs. S5, S6, and S14). Furthermore, PHP was blocked in animals with motoneuron-specific OAMB knockdown. While this block may partially reflect elevated baseline transmission (Fig. 7), it nonetheless suggests that the elements of the pathway mediating monoaminergic potentiation are shared with ones inducing or expressing PHP.

A picture emerges where slowed diffusion and nanoscopic protein compaction coincide with potentiation. The mechanism remains unclear, but one idea is that freezing Ca^2+^ channels, scaffolds, and vesicle sites in place enforces tight coupling for Ca^2+^-triggered release (9, 44). Alternatively, nanoscale Unc13 clustering may enhance its role in vesicle tethering (67). Based on our estimates (~10 Unc13 molecules per AZ; *SI Appendix*, Fig. S6) and the low number of functional release sites (42), vesicle capture is likely limiting—especially if multiple Unc13s must cooperate per site. While our imaging tracks both Unc13A and B, Bakshinska et al. (34) suggest octopamine acts specifically via Unc13A, consistent with our finding that Unc13A knockdown blocks potentiation (Fig. 3). We speculate that reduced Unc13A levels upon knockdown triggers compensatory recruitment of all remaining Unc13A into an active state, preventing further potentiation. If Unc13A is the key isoform, effective Unc13 molecule numbers per AZ may be even lower, making vesicle capture a rare event. In this case, compaction becomes functionally relevant to increase local Unc13A density (Fig. 2 *O* and *Q*), making the engagement of multiple tether proteins more likely. (M)Unc13 has been proposed to energetically stabilize vesicle priming, and we propose that the potentiation seen here reflects an increased readily releasable pool consistent with this role. Whether such changes yield ultrastructural alterations visible by electron microscopy (EM) remains unknown.

We used the *D. melanogaster* NMJ as a model system owing to its unmatched accessibility for live single molecule imaging, genetic manipulation, electrophysiological recordings, and behavioral profiling. However, due to the fundamental role of the PLC pathway, we predict that the fast potentiation mechanism described here—involving GPCR signaling, DAG production, and Unc13 compaction—holds relevance throughout the nervous system and across species. Indeed, α1-adrenergic receptors, mammalian counterparts of OAMB, are implicated in plasticity, cognition, and neurodegenerative disease (68). Future work based on the insight provided here could test the generality of this mechanism and its intersection with human disease.

## Materials and Methods

### Fly Husbandry, Stocks, and Handling.

Please see Key Resource Table (*SI Appendix*, Table S1) above for full details of these strains. Fly strains were maintained under standard laboratory conditions (69) and raised at 25 degrees Celsius. For experiments, male and female third-instar larvae were used unless otherwise stated. The genotypes utilized included Wild-type: +/+ (w^1118^), sourced from the Bloomington Drosophila Stock Center and WellGenetics; *OK6-Gal4* (70); *UAS-Unc13A-RNAi* (43); *UAS-OAMB-RNAi* (71); *UAS-syt-mScarlet-GCaMP8f* (41); *UAS-PLC-RNAi* (72); Unc13 Biosensor (43); *Unc13A C2B^K1862W^* (this paper); *Unc13A C1^H1723K^* (this paper); *Unc13mOES3.2* (this paper); *Unc13C1^H1723K^ mOES3.2* (this paper).

Genotypes:

Wildtype (*w^1118^*)

OAMB KD *(w^1118^;OK6-Gal4/+;UAS-OAMB-RNAi/+;)*

Syt::GCaMP8f (*w^1118^*; *OK6-Gal4/+;UAS-syt-mScarlet-GCaMP8f/+)*

ctrl (OAMB-RNAi) (*w^1118^*; *+/+*;*UAS-OAMB-RNAi/+)*

OK6-GAL4>UAS-syt-mScarlet-GCaMP8f(*w^1118^;OK6-Gal4/+;UAS-syt-mScarlet-GCaMP8f/+)*

Ctrl Genotype *(w^1118^;UAS-PLC-RNAi/+;+/+)*

PLC KD *(w^1118^;UAS-PLC-RNAi/OK6-Gal4;+/+)*

Unc13mEOS3.2 (*Unc13mEOS3.2*)

Ctrl(UAS-Unc13A-RNAi) (*w^1118^*;+/+;*UAS-Unc13A-RNAi/+*)

Unc13A KD (*w^1118^; OK6-Gal4/+; UAS-Unc13A-RNAi/+)*

OK6-GAL4>UAS-Δ-N-term-Unc13-GFP(*w^1118^;OK6-Gal4/OK6-Gal4;UAS-Δ-N-term-Unc13/UAS-Δ-N-term-Unc13*)

OAMB KD (OK6-GAL4>UAS-Δ-N-term-Unc13-GFP/UAS-Oamb-RNAi) (*w^1118^;OK6-Gal4/+;UAS-Δ-N-term-Unc13/UAS-OAMB-RNAi*

Unc13-C2B^KW^ (*w^1118^*;;;*Unc13-C2B^K1862W^*/ *Unc13-C2B^K1862^)*

Unc13-C1^HK^(*w^1118^*;;;*Unc13-C^H1723K^/ Unc13-C^H1723K^)*

Unc13-C1^HK^mEOS3.2(*Unc13-C1^H1723K^mEOS3.2*)

### Fly Mutagenesis Unc13 C1^HK^ and Unc13-C2B^KW^.

The Unc13-C1^HK^ mutation was based on the analogous mouse Munc13-1 mutant, where histidine 567 was replaced by lysine (14). Sequence alignment identified histidine 1723 as corresponding *Drosophila* Unc13A residue. The C1 domain is also present in the Unc13B splice isoform and this gene editing approach modifies both isoforms. CRISPR-mediated mutagenesis was conducted by WellGenetics Inc. following modified methods from Kondo and Ueda (73) to introduce a targeted break in *unc-13*/CG2999. For repair, A PBacDsRed cassette containing two PBac terminals and 3xP3-DsRed and two homology arms with point mutations (one inducing the amino acid change and one introducing a TTAA sequence to recognize proper integration on the other homology arm) was employed. Initial screening of F1 progeny used the DsRed marker to identify successful integration. The marker was excised and PCR plus sequencing of genomic DNA confirmed the presence of the desired point mutation and complete cassette removal in the final *Drosophila* line (74). Lines were validated using genomic PCR and sequencing methods to verify PiggyBac transposition alleles of w[*];;; unc-13-PA H1723K CRISPR[PBacDsRed]/In(4)*ciD, ciD panciD* fly by testing whether the selection marker(3XP3-DsRed) is precisely excised, the HK mutation present and an integrated TTAA sequence left behind in the coding exon of *unc-13/CG2999.* For the Unc13-C1^HK^ mutant, the forward primer 5′-CCGCTACAAAACGAAATGCT targeting the upstream homology arm the reverse primer, 5′-GTCCATAACAAAAAATTCTT, targeting the downstream homology arm were used to verify presence of the mutation.

### Generation of on-Locus Tagged Unc13-mEOS3.2 and Unc13-C1^HK^-mEOS3.2.

Briefly, C-terminally tagged Unc13mEOS3.2 was generated using a scarless CRISPR/piggyBac-based approach (https://flycrispr.org/). Tag mEOS3.2 and an assigned linker were knocked in right before the Stop Codon of *unc-13*, at the same stable integration site as the previously published Unc13-GFP construct (43). Tagging of the Unc13-C1HK mutant was achieved by employing the same strategy on the w[*];;; unc-13 PA H1723K CRISPR / In(4)ciD, ciD panciD strain.

The transgenic flies were produced by Well Genetics Inc. (Taipei City, Taiwan), following adapted protocols from Kondo and Ueda (73). Specifically, these lines were generated through CRISPR-Cas9–mediated genome editing via homology-dependent repair, employing a single guide RNA and a double-stranded DNA plasmid donor. Presence of the mEOS3.2 sequence in the final fly line was confirmed using the primer 5’- TGAGATGGCTTTGTTGCTTG in the mEOS3.2 sequence and 5’- GTGTGTGGGTGCGCATTTTA on the downstream homology arm.

### Tissue Preparation and Immunohistochemistry for Confocal Microscopy.

Third-instar larvae were dissected and stained as previously described (75). Larvae were incubated in 30 µL of HL3 containing either 20 µM octopamine for 1 min, 2 µM PMA for 10 min, 20 µM Philanthotoxin for 10 min, or DMSO (0.1%) for 1 min or 10 min before fixation in 4% paraformaldehyde in phosphate buffer saline (PFA, HistoLab, Askim, SE, HL96753.1000). Primary antibodies used: rabbit-Unc13A N-term [1:250, (44)] or rabbit anti-GFP (1:250, Thermo Fisher Scientific, MA, USA, A11122; RRID: AB_221569) or guinea pig-Unc13A N-term [1:250, (44)], or guinea pig-Unc13A (1:2,000, Absea Biotechnology GmbH, Beijing, China), and mouse-nc82 (1:250, Developmental Studies Hybridoma Bank [HSDB], University of Iowa, Iowa city, IA, USA; RRID: AB_2314866). Secondary antibodies used: goat anti-rabbit Alexa Fluor 488 (1:500, Thermo Fisher Scientific, MA, USA, A11008; AB Registry ID: AB_143165), or Alexa Fluor 488 goat anti-guinea pig (1:500, Thermo Fisher Scientific A11073; RRID: AB_2534117), goat anti-mouse CY3 (1:500, Jackson ImmunoResearch, PA, USA, 115-167-003; AB Registry ID: AB_2338709), goat anti-HRP Alexa Fluor 647 (1:250), Jackson ImmunoResearch, PA, USA, 123-605-021; AB Registry ID: AB_2338967).

#### Confocal imaging.

Confocal imaging was performed with an Abberior Infinity Line confocal and three-dimensional Stimulation Emission Depletion superresolution microscope. A Plan-achromat oil immersion objective (Olympus) with 63× 1.4 NA and a 0.19 mm working distance was used. The temperature of the room was maintained around 22 °C. All confocal images observed segments A3-A5 muscle 4 NMJs were visualized in live mode to adjust excitation laser channels for Alexa647, CY3, and Alexa488. A stack of images was collected at different z positions 300 nm apart to capture the entirety of the NMJ, the resolution would be set to 90 nm pixel size with the accumulation set to 3, and 3 NMJs per animal were taken.

#### Analysis of AZ morphology of confocal images.

Confocal stacks were processed in ImageJ (v. 2.14.0/1.54f) using the same analysis as described previously (44).

#### Line-profile analysis of Unc13-GFP fragment confocal images.

ImageJ was used to process Unc13-GFP confocal images to determine the highest gray value intensities in the middle of bouton using live line-profiles in single confocal slices. The highest gray values from the edge of bouton were averaged and divided by the lowest gray value in cytosol of bouton to generate a plasma membrane/cytosol ratio. Boutons were averaged across NMJs.

### Electrophysiology: Setup and Data Acquisition.

#### Current clamp recordings.

All current clamp experiments were performed according to ref. 45. Following changes were made: HL3 solution [low Mg2+ HL3 (5): 10 mM MgCl_2_], mEPSPs were recorded for 30 s and eEPSP responses were elicited by 5 stimulation pulses (300 µs, 8 V, 0.1 Hz). Current clamp recordings were performed at 0.4 mM Ca^2+^ (exception: Fig. 1*F* = 0.2 mM Ca^2+^, *SI Appendix*, Fig. S1 *A*–*C* = 0.3 mM Ca^2+^, and *SI Appendix*, Fig. S10 = 0.1 mM Ca^2+^).

Electrophysiological current clamp measurements, as well as data analysis were obtained following (45).

#### TEVC.

Electrophysiological TEVC experiments as well as TEVC recordings of baseline transmission and short-term plasticity in Unc13-C1^HK^ mutants were conducted following (45).

#### Octopamine and DMSO—electrophysiology.

After the initial current clamp recording, the same larvae were treated for 1 min with 0.1% DMSO (stored at –20 °C) or 1 min with 20 µM octopamine (stored at –20 °C as a 20 mM stock in DMSO). The fold-change in eEPSP amplitudes were calculated by dividing the post–octopamine treatment eEPSPs by the baseline eEPSPs, enabling quantification of the relative changes nduced by octopamine.

#### PLC inhibition—electrophysiology.

Larvae were treated for 1 min with 1 µM PLC inhibitor (Tocris Bioscience, U73122; freshly prepared as a 1 mM stock in DMSO) or with 1 µM PLC control drug (Tocris Bioscience, U73343; freshly prepared as a 1 mM stock in DMSO). Subsequently, mEPSPs and eEPSPs were recorded following octopamine treatment in current clamp.

#### Phorbol ester—electrophysiology.

For PMA experiments, 2 µM PMA (2 mM stock in DMSO, stored at –20 °C) or the same volume of DMSO for control experiments was mixed with Ca^2+^-free HL3. 40 µL of the respective solution was incubated on the opened filet for 10 min at room temperature and immediately afterward three times rinsed with Ca^2+^-free HL3 before transferred to the bath chamber. TEVC recordings were performed as described above in HL3 with 0.4 mM Ca^2+^.

#### PHP—electrophysiology.

PHP experiment was performed as in ref. 45, except extracellular Ca^2+^ concentrations of 0.4 mM was used.

### Single-Particle Tracking PALM.

Live sptPALM experiments were performed on male third-instar larvae of Unc13-mEOS3.2 and/or Unc13-C1^HK^-mEOS3.2 flies at 25 °C. Larval body wall preparation for single particle tracking were prepared according to previously described protocols (9, 76). Imaging experiments were carried out using an inverted total internal reflection fluorescence (TIRF) setup. The microscope (Nikon Eclipse Ti) has a 100x NA 1.49 Apo TIRF oil objective (Nikon). Up to 10,000 images were recorded at a frame rate of 20 Hz using an EMCCD camera (iXon+ 897, Andor Technology) controlled by NIS-Elements (Nikon). A 1.6 magnification lens was employed to achieve a final pixel size of 107 × 107 nm.

#### Live acute 1-min drug application: sptPALM imaging before and after drug (octopamine, PMA).

Male larvae expressing endogenously tagged Unc13-mEOS3.2 and/or Unc13-C1HK-mEOS3.2 were dissected in Ca^2+^/Mg^2+^-free HL3.1 saline (in mM: NaCl 70, KCl 5, NaHCO_3_ 10, Sucrose 115, Trehalose 5, HEPES 5). Larvae were live-stained for 5 min with HRP-488 in HL3.1 imaging buffer (0.4 mM Ca^2+^, 10 mM Mg^2+^, DMSO), then briefly rinsed in the same buffer.

Imaging began with an internal control NMJ (type 1b, Muscle 4, segments A2–A4) at the HRP Z-plane. A second, similarly located NMJ was then brought into focus. Prior to imaging, the DMSO buffer was replaced with HL3.1 imaging buffer containing 20 µM octopamine or 2 µM PMA, perfused under the filet for 30 s. Live sptPALM imaging of Unc13 at muscle 4 Ib boutons was conducted for 8.5 min per NMJ at 20 Hz.

#### 10-min drug application, 20-min sptPALM imaging (PhTx).

For PhTx experiments, Unc13-mEOS3.2 larvae were filleted and exposed for 10 min to either 50 µM PhTx or control HL3.1 buffer. After a 5-min HRP-488 stain and wash, larvae were stabilized for 5 min in HL3.1 buffer (4 mM Mg^2+^, 1.5 mM Ca^2+^) before imaging muscle 4 Ib NMJs. This protocol follows our previously published method (9).

#### SptPALM analyses.

For each sptPALM experiment, Unc13mEOS3.2 raw data were ImageJ-NanoJ drift corrected and analyzed in three parts a) stepwise bleaching of the Unc13 attached fluorophore mEOS3.2 to generate relative Unc13 molecule count b) motility analysis c) signal distribution and density.

Bleach curve analysis: Localization and trajectory reconnection of Unc13mEOS signals were performed using a wavelet-based and simulated annealing algorithm, accounting for molecule position and intensity, as our previously published method (9).

#### Motility analysis.

Diffusion coefficients (D) were obtained by exponential fitting of the first eight MSD plot points. MSDs from fixed samples indicated that molecules with D ≥ 0.002 μm^2^/s are mobile under our conditions. Exponential fitting also determined the confinement radius; trajectories with radii outside 70 to 800 nm were excluded. Individual Unc13 tracking followed these parameters using the PALMtracer plugin in Metamorph.

#### Tessellation analysis.

Tessellation analysis followed methods from our previous work on live endogenous Cacophony localization (9), which is built on localization criteria from GFP-tagged Cacophony (dSTORM) (6), and Unc13 (dSTORM) (53). Briefly, Unc13 localizations from drift-corrected live sptPALM movies (mutant/treated and controls) were processed by cluster analysis based on Voronoï tessellation software SR-Tesseler. ThunderSTORM extracted PALM localization maps, were further analyzed with SR-Tesseler software to visualize Unc13 spatial distribution. Filtering parameters used below identified high-density Unc13 localizations at AZs under optimal TIRF Z-focus, excluding out-of-focus or side-view synapses. Tessellation parameters for AZ boundaries were Voronoi object density factor 100 to 200, minimum area 2,000 nm^2^, and minimum localization count 100 to 200. Nanocluster (NC) boundary settings were density factor 1.5, minimum area 50 nm^2^, and localization count ≥20. Clusters were filtered to exclude those lacking nanoclusters or falling outside AZ/NC size ranges (AZ: 600 to 20 nm, NC: 300 to 20 nm). Additional criteria included localization limits (3000/AZ, 3000/NC), area limits (AZ: 50,000 nm^2^, NC: 7,000 nm^2^), and density thresholds (≥1.5 localizations/nm^2^ for both AZ and NC). These settings reflect prior dSTORM and QPAINT studies and our own prior work, to accurately define Unc13 nanodomains at the *Drosophila* NMJ presynaptic membrane (53).

### Analysis of AP-Induced Presynaptic Ca^2+^ Levels.

To estimate AP-induced changes in presynaptic Ca^2+^ levels, the fluorescent Ca^2+^ sensor GCaMP8f fused to mScarlet and Synaptotagmin (40) was expressed using OK6-Gal4. Larval dissection and nerve stimulation were performed as for electrophysiological recordings. Imaging was done on muscle 6/7 NMJs using epifluorescence microscopy with Light Emitting Diode (LED) light source and scientific Complementary Metal–Oxide–Semiconductor (sCMOS) camera. Experiments were performed in HL3 containing 0.4 mM Ca^2+^ and 0.1 mM spermine to block muscle contraction. NMJs were imaged before and 1-min after octopamine (20 µM) or mock treatment. Images were acquired at 20/5 Hz with 50/100 ms exposure times for experiments with 1/20 APs (20APs@20Hz) (*SI Appendix*, Fig. S1 *D* and *E*).

Analysis used Fiji-ImageJ with manually selected ROIs around basal fluorescence. Background subtraction was performed in each frame. The relative changes in fluorescence were quantified (ΔF/F_0_) where F_0_ is the mean prestimulation fluorescence (six frames before stimulation) and ΔF = F(t) − F_0_ (F(t) is fluorescence at time t). Maximal ΔF per NMJ was paired-compared before/after treatment. Outlier signals were excluded using the ROUT Method (Q = 1%) in GraphPad Prism. Only recordings with visible changes were used. Please refer to the key resource table for details regarding equipment and reagents.

### Crawling Assay.

Third-instar larvae were grown at 25 °C/65% humidity in 12 h light/dark cycles at low density in standard food vials. Experiments were conducted at 22 °C with individual larvae placed in 14 cm petri dishes filled with 1% agarose. Crawling behavior was recorded for 1 min at 15 frames/s using PiVR (77). Larvae were then maintained in food-free agar for 2 h before retesting. Trajectories were centered for display, crawling speed computed using scripts from ref. 77, and statistical analysis performed in R (78).

## Supplementary Material

Appendix 01 (PDF)

## Data Availability

Figure source data are published in the supporting information (*SI Appendix*, Tables S2 and S22). The dataset has also been deposited in Zenodo and will be made publicly available upon publication (https://doi.org/10.5281/zenodo.15629377) (79). No new software codes were developed in this project (references to all used published codes are listed in *SI Appendix*, Table S1).
