# Supplementary material for: Monoamine-induced diacylglycerol signaling rapidly accumulates Unc13 in nanoclusters for fast presynaptic potentiation
Source: Proc Natl Acad Sci U S A. 2025 Aug 20;122(34):e2514151122. doi: 10.1073/pnas.2514151122 (PMC12403152; doi:10.1073/pnas.2514151122)
Supplement: Supplementary file 1 — Appendix 01 (PDF) [file pnas.2514151122.sapp.pdf]

PNAS

Supporting Information for
Monoamine-induced diacylglycerol signaling rapidly accumulates Unc13 in nanoclusters for fast presynaptic potentiation

Natalie Blaum*, Tina Ghelani*, Torsten Götz*, Keagan S. Chronister*, Mercedes Bengochea, Livia Ceresnova, Christian F. Christensen, Thiago C. Moulin, Hanna Kern, Ulrich Thomas, Martin Heine, Stephan J. Sigrist, and Alexander M. Walter#

Alexander M. Walter
Email: awalter@sund.ku.dk

This PDF file includes:

Figures S1 to S14
Tables S1 to S22
SI References

Supporting Figures

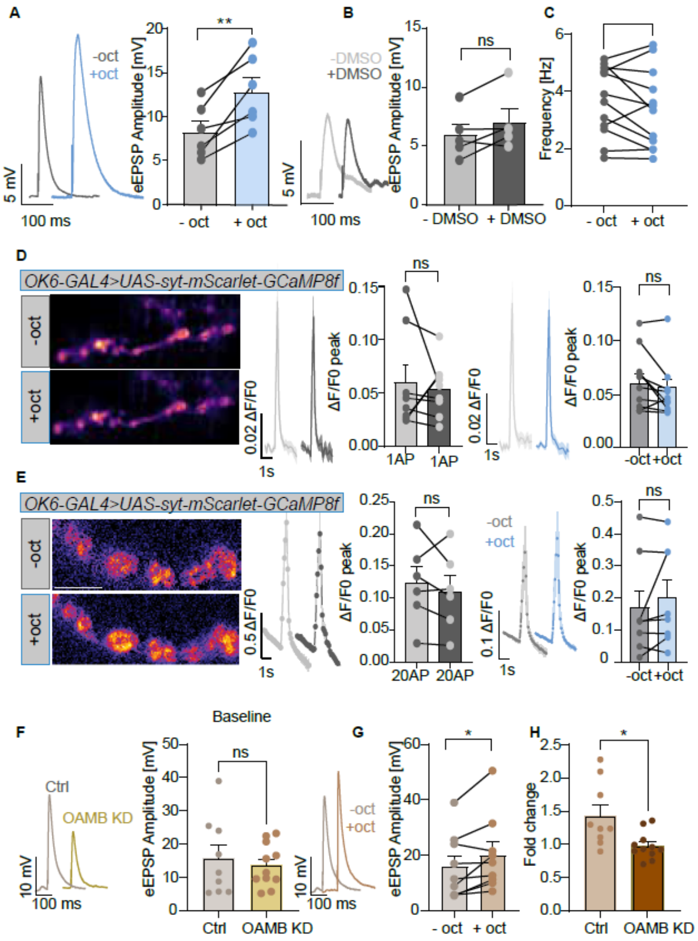

Fig. S1: Control experiments, establishing that treatment protocol does not affect synaptic responses, analysis of AP-induced Ca^{2+} changes, genotype comparison for OAMB KD, and control experiment on OAMB control genotype showing octopamine potentiation.

(**A, B, C, F, G, H**) Analysis of current clamp recordings (muscle 6 NMJs, (**C, F, G, H**) 0.4 mM or (**A** and **B**) 0.3 mM extracellular Ca^{2+}) of AP-evoked synaptic activity. (**A**) Representative AP-evoked eEPSP responses (average of five repetitions in one cell) from wildtype synapses (w1118) before (light grey) and after 1-minute 20 µM octopamine incubation (blue) and quantification of eEPSP amplitudes. (**B**) Representative AP-evoked eEPSP responses (average of five repetitions in one cell) from wildtype synapses (w1118) before (grey) and after 1-minute DMSO incubation (dark grey) and quantification of eEPSP amplitudes. (**C**) Analysis of current clamp recordings (muscle 6 NMJs, 0.4 mM extracellular Ca^{2+}) of the frequency of spontaneous activity (mEPSP) in wildtype animals before (-oct, grey) and one minute after (+oct, light blue) 20 µM octopamine treatment. (**D-E**) Representative images and fluorescence changes over time from either muscle 6/7 (**D**) or muscle 4 (**E**) presynaptic terminals (OK6-Gal4>UAS-syt::mScarlet::GCaMP8f) in response to one (**D**) or 20 action potentials delivered at 20 Hz (**E**) (0.4 mM Ca^{2+} in the external medium). In control experiments, no treatment was administered and the responses compared to a first (light grey) and second (dark grey) stimulation 1 minute apart. The effect of octopamine treatment was investigated in separate animals by comparing fluorescence changes before (grey) and 1 minute after 20 µM octopamine treatment (blue). Bar plots illustrate animal-wise quantification of maximal ΔF/F0 values with paired comparison of the peak florescence signal before and after treatment. (**F**) Right: representative AP-evoked eEPSP responses (average of five repetitions in one cell) from Ctrl

(UAS-OAMB-RNAi, grey) and OAMB KD (OK6-Gal4>UAS-OAMB-RNAi, yellow) mutant synapses and quantification of eEPSP amplitude. (**G**) Representative AP-evoked eEPSP responses (average of five repetitions in one cell) from control synapses (UAS-OAMB-RNAi) before (grey) and after 1-minute 20 µM octopamine incubation (beige) with quantification of eEPSP amplitudes. (**H**) Comparison of the fold-change in eEPSP amplitudes after 1 minute of octopamine incubation divided by the eEPSP amplitudes prior to treatment for the control condition (UAS-OAMB-RNAi, beige) and OAMB KD animals (OK6-Gal4>UAS-OAMB-RNAi, brown). Number of cells (n) and animals (N) investigated: n/N(Octopamine, A) = 6/6; n/N(DMSO, B) = 5/5; n/N (w1118 frequency, C) = 12/12; n/N(OK6-Gal4>UAS-syt-mScarlet-GCaMP8f, E) = 6/6; n/N(Ctrl, F,H) = 9/9; n/N(OAMB KD, G,H) = 11/11. For exact genotypes see methods. Data depicts mean values ± SEM. Statistical analysis with paired parametric t-tests (**A, B, C, D, E, G**) or Mann-Whitney test (**F, H**). For details see table S3. n.s., p > 0.05; *p % 0.05; **p % 0.01. Scale bar: 10 µm.

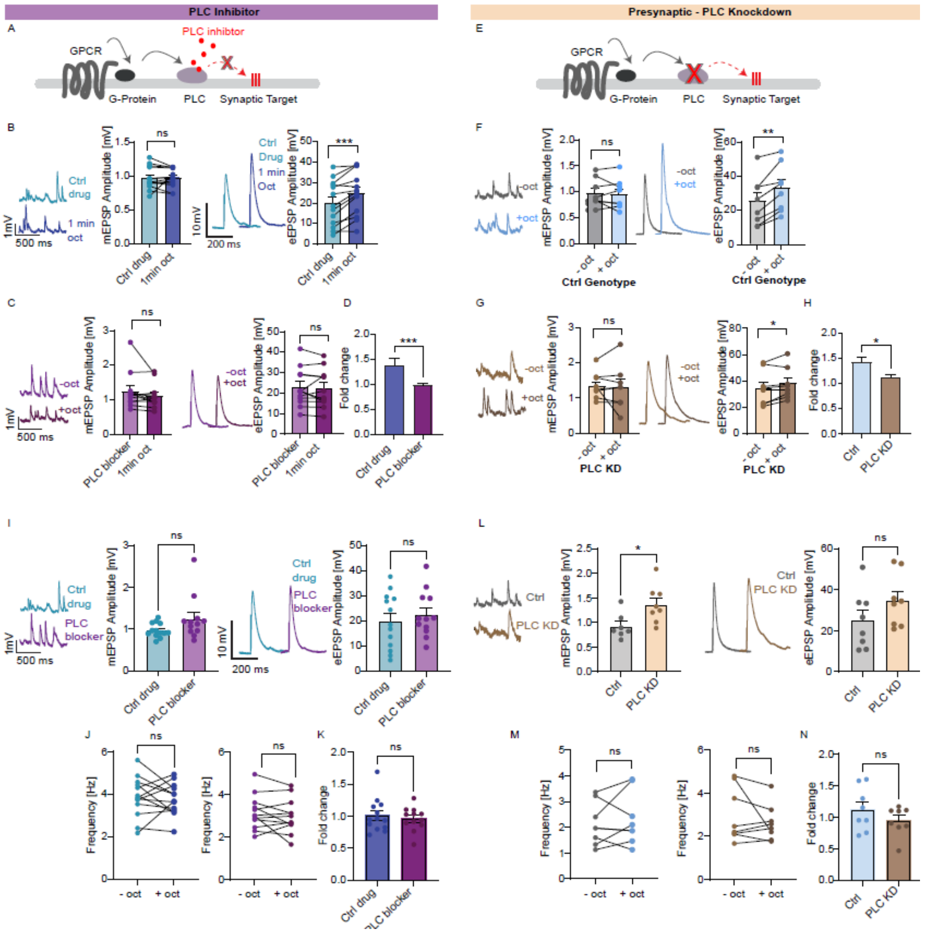

Figure S2: Additional data for the experiment establishing that octopamine-induced potentiation requires presynaptic phospholipase C. (**A** and **H**) Scheme of phospholipase C signaling pathway indicating a G protein-coupled receptor (GPCR) g-protein, Phospholipase C (PLC) and diacylglycerol (DAG) and the PLC inhibitor blocking PLC (**A**) or the presynaptic PLC knockdown (**H**). (**B**, **C**, **E**, **I**, **J**, **L**) Analysis of current clamp recordings (muscle 6 NMJs, 0.4 mM extracellular Ca^{2+}) of spontaneous (left) and AP-evoked synaptic activity (right). (**B**) Left: representative example traces of spontaneous mEPSPs from wildtype animals (w1118) with PLC control drug (U73343, 1 µM; light blue) and after 1 min 20 µM octopamine (dark blue) and quantification of mEPSP amplitudes after PLC ctrl drug before octopamine. Right: representative AP-evoked eEPSP responses (average of five repetitions in one cell) wildtype animals (w1118) with 1-minute PLC ctrl drug incubation (U73343, 1 µM; light blue) and followed by 1-minute incubation with 20 µM octopamine (dark blue) and quantification of eEPSP amplitudes. (**C**) Left: representative example traces of spontaneous mEPSPs from wildype animals (w1118) with PLC inhibitor (U73122, 1 µM; light purple) and followed by 1-minute incubation with 20 µM octopamine (dark purple) and quantification of mEPSP amplitudes with PLC inhibitor and before octopamine (light purple) and after 1-minute incubation with 20 µM octopamine (dark purple). Right: representative AP-evoked eEPSP responses (average of five repetitions in one cell) in wildtype animals (w1118) with 1 minute PLC inhibitor (U73122, 1 µM; light purple) and followed by 1-minute incubation with 20 µM octopamine (dark purple) and quantification of eEPSP amplitudes (Panel C (right) reused from Fig. 1D). (**D**) Comparison of the fold change in eEPSP amplitudes after and before 1 minute of octopamine incubation between control drug (blue) and PLC inhibitor (purple). (**I**) Left: representative example traces of spontaneous mEPSPs from control genotype synapses (Ctrl genotype, *UAS-PLC-RNAi*) before and after 1-minute octopamine incubation (-oct, grey; +oct, blue) and quantification of mEPSP amplitudes. Right: representative AP-evoked eEPSP responses (average of five repetitions in one cell) from control genotype synapses and quantification of eEPSP amplitude (oct, grey; +oct, blue). (**J**) Left: representative example traces of spontaneous mEPSPs

from PLC knockdown synapses (*OK6-Gal4>UAS-PLC-RNAi*) before octopamine incubation (beige, top) and after 1-minute incubation with 20 μM octopamine (brown, bottom) together with a cell-wise quantification of mEPSP amplitudes before (-oct, beige) and 1 min after incubation with 20 μM octopamine (+oct, brown). Right: representative AP-evoked eEPSP responses (average of five repetitions in one cell) from PLC knockdown synapses and quantification of eEPSP. (Panel J(right) reused from Fig. 1E) (**K**) Comparison of the fold change in eEPSP amplitudes after 1 minute of octopamine incubation between Ctrl genotype (blue, *UAS-PLC-RNAi*) and PLC knockdown (brown, *OK6-Gal4>UAS-PLC-RNAi*) animals. (**E**) representative example traces of spontaneous mEPSPs after 1- minute control drug incubation (1 μM, blue, top) and after 1-minute PLC inhibitor treatment (1 μM, purple, bottom) together with a cell-wise quantification of mEPSP amplitudes after 1-minute control drug (blue) and PLC inhibitor (purple) incubation. Right: representative AP-evoked eEPSP responses (average of five repetitions in one cell) from wildtype synapses with displayed drug treatment and quantification of eEPSP amplitudes after indicated drug treatment (ctrl drug=blue; PLC inhibitor=purple). (**F**) Quantification of mEPSP frequency in control condition (left) PLC inhibitor condition before and after one-minute 20 μM octopamine incubation (right) with the comparison of the fold change in mEPSP frequency between control drug and PLC blocker (**G**). (**L**) Left: representative example traces of spontaneous mEPSPs from Ctrl (*UAS-PLC-RNAi*, grey, top) and PLC knockdown (*OK6-Gal4>UAS-PLC-RNAi,* beige, bottom) synapses and quantification of mEPSP amplitudes. Right: representative AP-evoked eEPSP responses (average of five repetitions in one cell) from Ctrl and PLC knockdown synapses and quantification of eEPSP amplitudes. (**M**) Quantification of mEPSP frequency in control genotype (left, *UAS-PLC-RNAi*) and PLC KD (*OK6-Gal4>UAS-PLC-RNAi*) before and after one-minute 20 μM octopamine incubation (right) with the comparison of the fold change in mEPSP frequency between control genotype and PLC KD (**N**). Number of cells (n) and animals (N) investigated: n/N: n/N(*Ctrl drug*) = 13/13, n/N(PLC inhibitor) = 12/12, n/N(Ctrl genotype) = 8/8, n/N(PLC knockdown) = 8/8. For exact genotypes see methods. Data depict mean values ± SEM. Statistical analysis with unpaired, Mann-Whitney U test or with paired parametric t-tests. For details see table S4. n.s., p > 0.05; *p % 0.05; **p % 0.01; ***p % 0.001.

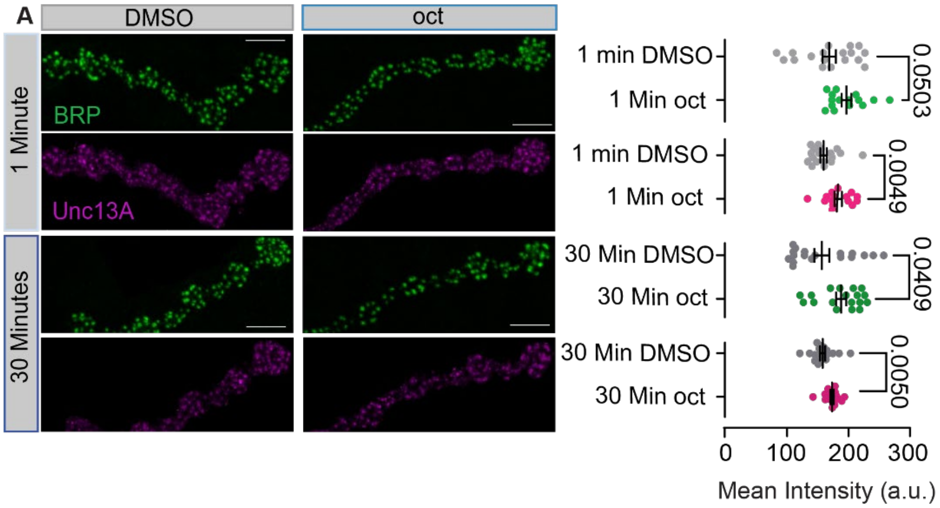

Figure S3: 1- and 30 minute octopamine treatment similarly increase BRP and Unc13A confocal signals. Left: representative confocal images of muscle 4 NMJs of segment A3-A5 from third-instar wildtype larvae (w1118) labeled with antibodies against BRP (green, top) and Unc13A (pink, bottom) either treated for 1 minute or 30 minutes with DMSO or octopamine in DMSO (20 µM). Right: Quantification of BRP and Unc13A puncta intensities under DMSO or octopamine incubation at 1 min and 30 minutes. Number of cells (n) and animals (N) investigated: n/N(DMSO 1 minute, **A**) = 18/6; n/N(oct 1 minute, **A**) =15/5; n/N(DMSO 30-minute, **A**) = 18/6; n/N(oct 30-minute, **A**) = 18/6. For exact genotypes see methods. Data depicts mean values ± SEM. Statistical analysis with unpaired t-tests, p-values as indicated. For details see table S5. Scale bar in (**A**): 5 µm

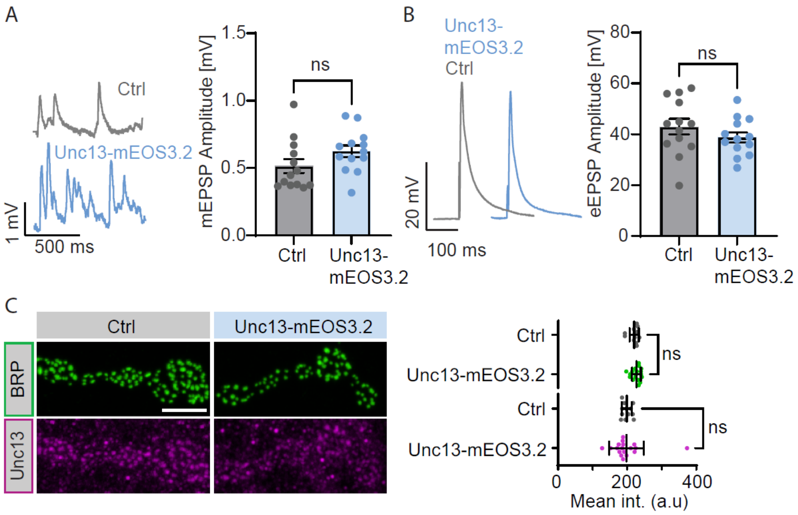

Figure S4: CRISPR Cas9 mediated mEOS3.2 tagging of the endogenous Unc13 protein does not affect baseline synaptic properties. (**A**) Representative example traces of spontaneous mEPSPs from wildtype control (Ctrl) animals (w1118; grey, top) and Unc13mEOS3.2 animals (blue, bottom) together with a cell-wise quantification of mEPSP amplitudes. (**B**) Representative AP-evoked eEPSP responses (average of five repetitions in one cell) from control synapses (grey) and Unc13mEOS3.2 animals (blue) and quantification of eEPSP amplitudes. (**C**) Representative confocal images (left) of muscle 4 NMJs from wildtype (Ctrl, w1118, grey) and Unc13mEOS3.2 (C, light Blue) stained with antibodies against BRP (green, top) and the C-terminal region (consensus for Unc13A and -B) of Unc13 (purple, bottom, see methods). Right: Quantification of mean intensities of BRP (top) and Unc13A (bottom) per NMJ. Number of cells (n) and animals (N) investigated: n/N(Ctrl, **A**) = 13/13; n/N(Unc13mEOS3.2, **B**) = 13/13; n/N(Ctrl, **C**) = 12/4; n/N(Unc13mEOS3.2, **B**) = 18/6. For exact genotypes see methods. Data depicts mean values ± SEM. Statistical analysis with unpaired t-tests. For details see table S7. n.s., $p > 0.05$. Scale bar in (**C**): 5 µm

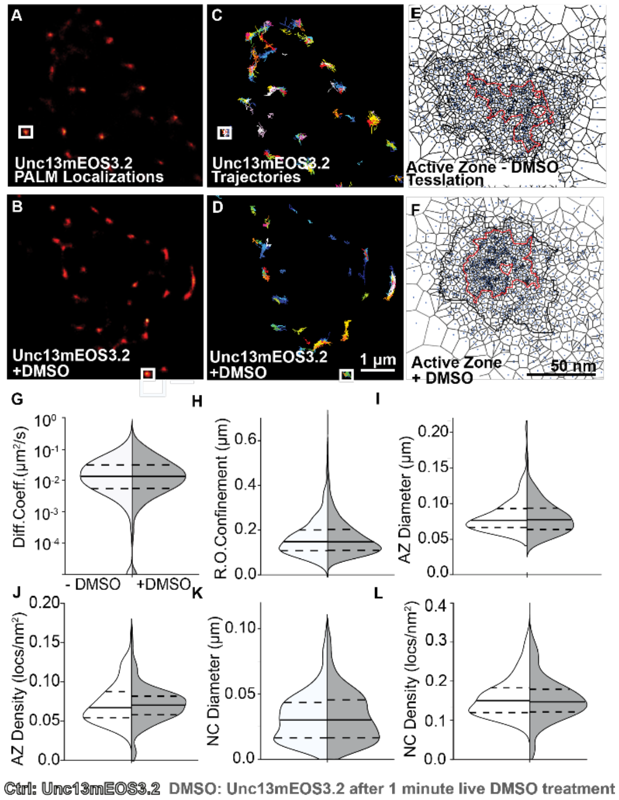

Figure S5: Live 1-minute application of DMSO does not impact Unc13 motility or nanoscale organisation. Live sptPALM imaging of Unc13mEOS3.2 at muscle 4 NMJs, performed in 0.4 mM Ca^{2+} and 10 mM Mg^{2+} containing HL3.1, before (Ctrl) and after a 1-minute incubation of DMSO in HL3.1 (DMSO). This control experiment was performed concomitantly with experimental conditions in Figure 2. Images show representative sptPALM recordings (**A** and **B**), trajectory maps (**C** and **D**), and tessellation analysis representations of Unc13mEOS3.2 before and after 1-minute DMSO treatment (**E**, and **F**). (**G** and **H**) Quantification of diffusion coefficients and radii of confinement from live Unc13 channel sptPALM imaging. (**I** to **L**) Tessellation analysis from the same sptPALM dataset was analyzed in (**F**) to (**I**) for diameters and densities of Unc13 localizations within AZ (**I**—**J**) and NC cluster (**K**—**L**) boundaries. Number of NMJs (n), animals (N), number of AZs (X) and number of individual trajectories (**Y**) investigated: n/N/X/Y(*Ctrl*, A) = 10/10/252/1437; n/N (DMSO, B) = 10/10/201/2966; Data depict mean values ± SEM. For details see table S8. Statistical significance is denoted as asterisks: $^{**}p < 0.01$, $^{***}p < 0.001$, and $^{****}p < 0.0001$. Data distribution was statistically tested with a Kolmogorov-Smirnov test. AU, arbitrary units. Scale bars, 1 μm (**A** to **D**) and 50 nm (**E** and **F**).

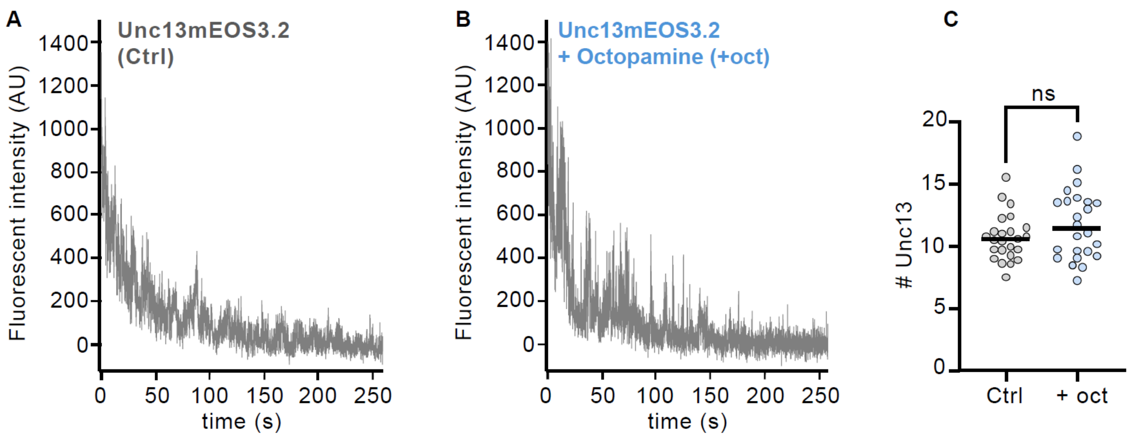

Figure S6: Molecular counting of Unc13mEOS3.2 sptPALM live imaging experiments in *Drosophila* larvae. Estimation of relative channel numbers based on the blinking fluorescent emission of mEOS3.2 tagged UNC13 molecules via bleach curve analysis. The average fluorescent intensity of individual synapses was recorded for 250s. To estimate signal bleach steps, fluorescent intensity measurements over time were normalized by first subtracting all intensity values to the average background signal and second to the average amplitude of single fluorophore events at the end of the recording. Bleach curve fluorescent intensity measurements of (**A**) Unc13mEOS3.2 controls (Ctrl, -oct), and (**B**) Unc13mEOS3.2 with octopamine (+ oct). (**C**) Quantification of relative Unc13 numbers from control and after octopamine. For comparison, quantification of relative Unc13 numbers extracted from a subset of the same data analyzed in live sptPALM experiments of Fig. 2. Number of NMJs (n) and animals (N) and number of AZs (X) investigated: n/N/X(Ctrl in ***C***) = *3*/3/24; n/N/X(+ oct in ***C***) = *3*/3/24. Data distribution was statistically tested with unpaired T-test. For details see table S9. N.s.: p>0.05.

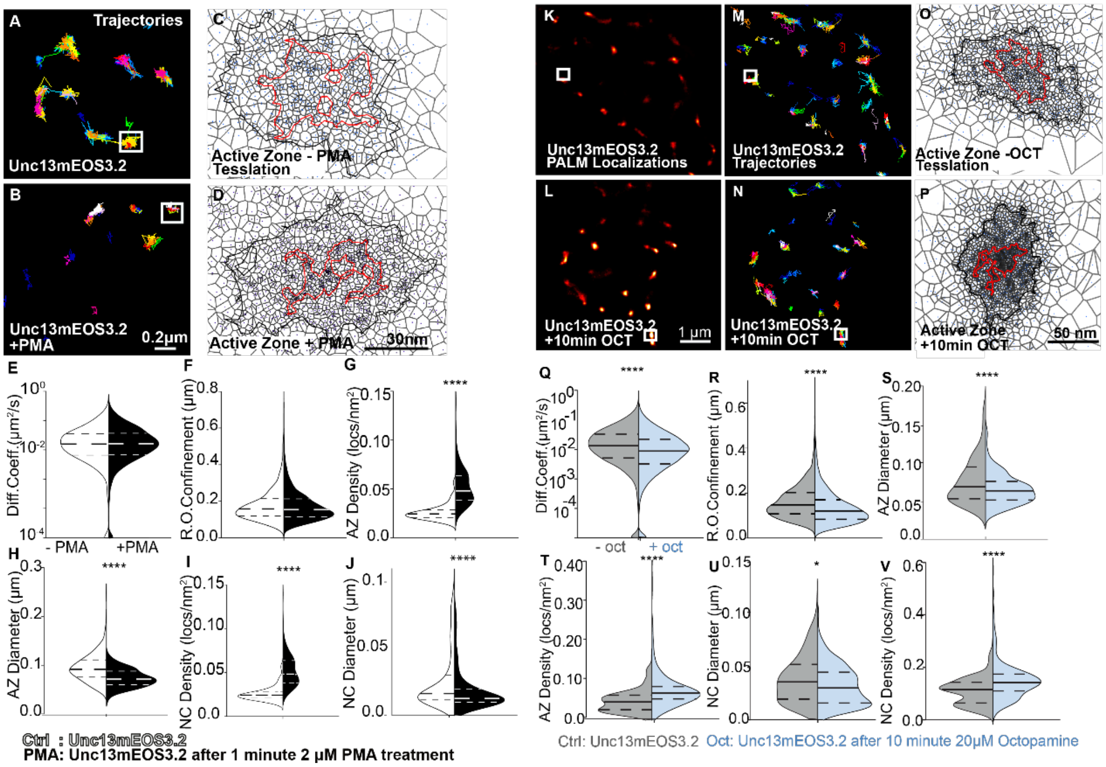

Figure S7: Live *in vivo* imaging of endogenously tagged Unc13 molecules undergoing a 10-minute octopamine treatment or 1-minute PMA treatment. Live sptPALM imaging of endogenously tagged Unc13mEOS3.2 at muscle 4 NMJs, performed in 0.4 mM Ca^{2+} and 10 mM Mg^{2+} containing HL3.1, before (Internal control/Ctrl) and after either a 1-minute 2 μM PMA incubation or a 10-minute incubation of 20 μM octopamine in HL3.1 imaging buffer. Images show representative sptPALM recordings depicting trajectory maps of boutons (**A-B**, **K-L**), and tessellation analysis representations (**C-D**, **M-N**) of Unc13mEOS3.2 AZS before and either 1-minute after PMA treatment or 10-minute octopamine treatment. (**E-F**, **O-P**) Quantification of diffusion coefficients and radii of confinement from live Unc13 channel sptPALM imaging. (**G-J, Q-T**) Tessellation analysis from the same sptPALM datasets for both experiments was analyzed for diameters and densities of Unc13 localizations within AZ (**G-H**) and NC cluster (**I-J**) boundaries. Number of NMJs (n), animals (N), number of AZs (X) and number of individual trajectories (Y) investigated: n/N/X/Y(Ctrl for PMA) = 8/8/251/2380; and n/N X/Y (1-min PMA) = 8/8/101/2931; n/N/X/Y(Ctrl for 10min Oct) = 18/18/287/5924; and n/N X/Y (10-min Oct) = 17/17/408/8747; Data depict mean values ± SEM. For details see table S10. Statistical significance is denoted as asterisks: **p < 0.01, ***p < 0.001, and ****p < 0.0001. Data distribution was statistically tested with a Kolmogorov-Smirnov test. AU, arbitrary units. Scale bars, 0.2 μm (A) and 30 nm (D).

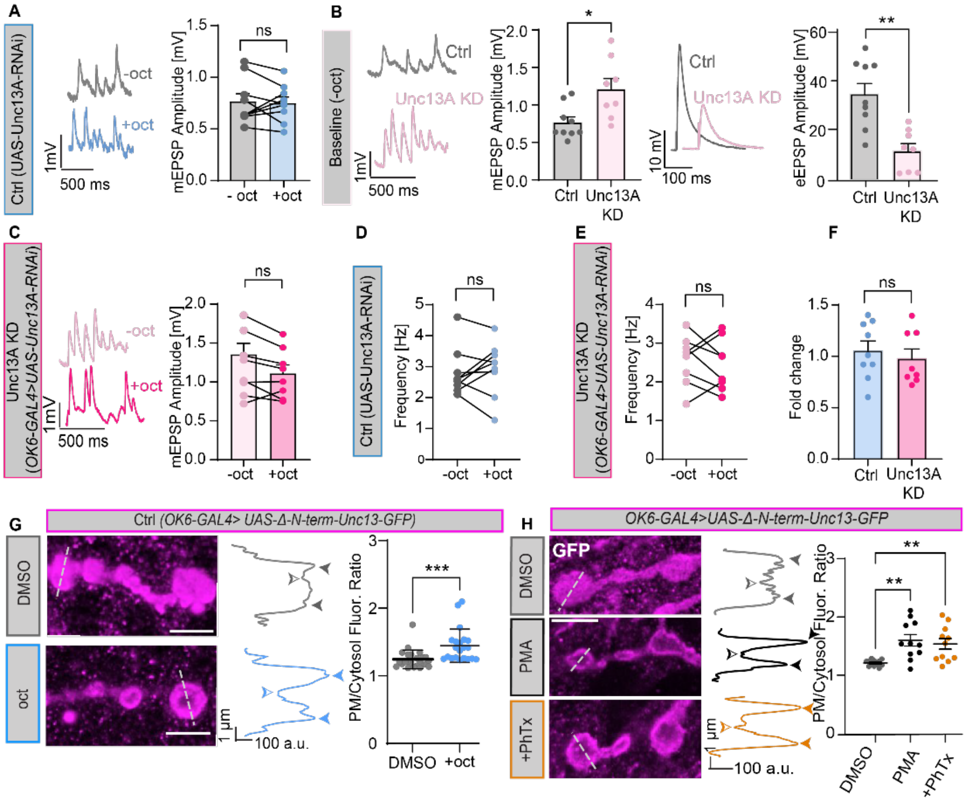

Figure S8: Additional electrophysiological characterization of Unc13A KD experiment and membrane binding analysis of the Unc13 C-terminal fragment. Analysis of current clamp recordings (muscle 6 NMJs, 0.4 mM extracellular Ca^{2+}). (**A**) Representative example traces of spontaneous mEPSPs from control animals (ctrl, *UAS-Unc13A-RNAi*) together with a cell-wise quantification of mEPSP amplitudes before (-oct, grey) and 1 min after incubation with 20 µM octopamine (+oct, blue). (**B**) Representative example traces of spontaneous mEPSPs and eEPSPs and cellwise quantification of mEPSP/eEPSP amplitudes from control (ctrl, *UAS-Unc13A-RNAi*) and Unc13A knock down (Unc13A KD, *OK6-Gal4>UAS-Unc13A-RNAi*) synapses. (**C**) Representative example traces of spontaneous mEPSPs from Unc13A KD synapses (Unc13A KD, *OK6-Gal4>UAS-Unc13A-RNAi*) before (light pink, top) and 1-minute after 20 µM octopamine incubation (pink, bottom) together with a cell-wise quantification of mEPSP amplitudes before (-oct, light pink) and 1 min after incubation with 20 µM octopamine (+oct, pink). (**D**) Analysis of mEPSP frequency in control animals (*UAS-Unc13A-RNAi)* before (-oct, grey) and one minute after 20 µM octopamine treatment (+oct, light blue). (**E**) mEPSP frequency before (-oct, light pink) and one minute after (+oct, pink) octopamine treatment in Unc13A KD (*OK6-Gal4>UAS-Unc13A-RNAi*) animals. (**F**) Comparison of the fold-change in mEPSP frequency after 1 minute of octopamine incubation divided by the mEPSP frequence prior to treatment for the control condition (*UAS-Unc13A-RNAi,* blue) and Unc13A KD animals (*OK6-Gal4>UAS-Unc13A-RNAi*, pink). (**G** and **H**) Left: Representative confocal images of GFP signal at muscle 4 NMJs (segment A3–5) presynaptically expressing the labelled Unc13 fragment. (**G**) Genetic control for Fig. 3F. Larvae were either incubated with DMSO alone (DMSO, 1 minute, grey), or with octopamine (oct, 20 µM, 1 minute, blue). GFP intensity profiles were collected at dashed lines. Middle: Line profiles from respective images representing Unc13-GFP fluorescent intensities and the ratio between highest and lowest intensities quantified (see methods). Right: Quantification of intensity ratios across NMJs. (**H**) Same as G, except larvae were incubated either with DMSO alone (DMSO, 10 minutes, grey), or PMA (PMA, 2 µM, 10 minutes, black), or philanthotoxin (+PhTx, 20 µM, 10 minutes, orange) Number of cells (n) and animals (N) investigated: n/N: n/N(Ctrl (UAS-Unc13A-RNAi), **A**) = 9/9; n/N(Unc13A KD, **C**) = 8/8n/N(Genetic ctrl, **D**) = 9/9; n/N(Unc13A KD, **E**) = 8/8; n/N(DMSO, **G**) = 24/8; n/N(oct, **G**) = 21/7, n/N(DMSO, OK6-Gal4>UAS-Δ-N-term-Unc13-GFP, **H**) = 12/4;

n/N(PMA, OK6-Gal4>UAS-Δ-N-term-Unc13-GFP, **H**) = 12/4; n/N(PhTx, OK6-Gal4>UAS-Δ-N-term-Unc13-GFP, **H**) = 11/4. For exact genotypes see methods. Data depicts mean values ± SEM. Statistical analysis with unpaired, Mann-Whitney U test (**F**), or with paired parametric t-tests (**D** and **E**), or unpaired t-test (**G**), or unpaired, one-way Anova (**H**). For details see table S12. n.s., p > 0.05; *p % 0.05; **p % 0.01; ***p % 0.001. Scale bar in (**G**, **H**): 5 µm

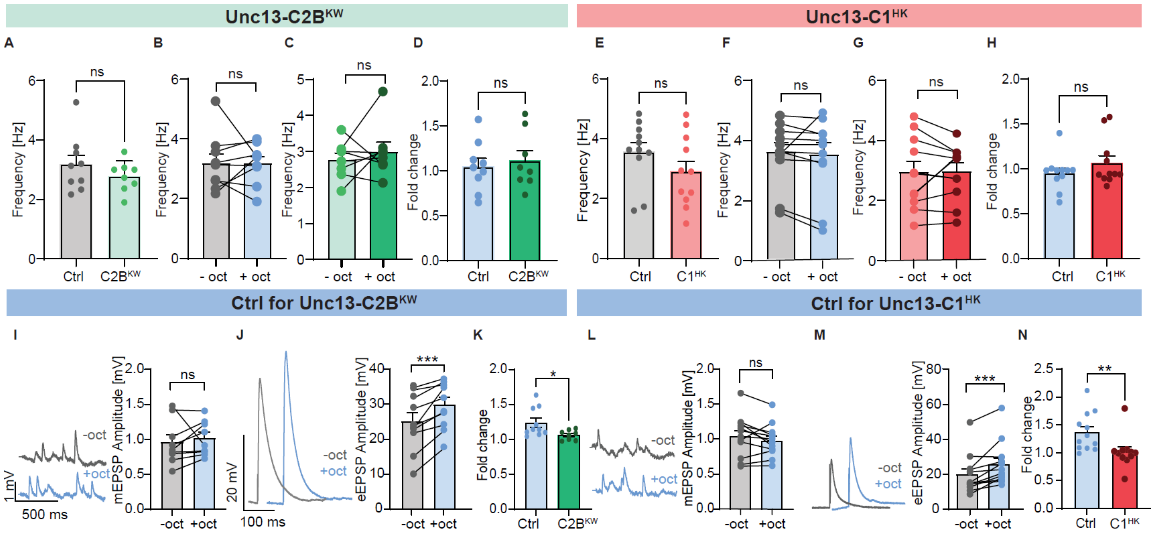

Figure S9: Further analysis for Unc13 C2B and C1 mutants and additional control experiments. (**A**) Comparison of current clamp recordings (muscle 6 NMJs, 0.4 mM extracellular Ca^{2+}) shows the frequency of spontaneous activity (mEPSP) in wildtype animals and Unc13-C2B^{KW} mutants before octopamine treatment**.** (**B**) Analysis of the frequency of mEPSP in wildtype animals before and after 1-minute 20 µM octopamine treatment. (**C**) Analysis of the frequency of mEPSP in Unc13-C2B^{KW} mutants before and after octopamine treatment. (**D**) Comparison of the fold change in mEPSP frequency after 1-minute of octopamine incubation between wildtype condition (blue, Ctrl, w1118) and Unc13-C2B^{KW} mutants (green). (**E**, **F**, **G**, **H**) Same analysis as in (**A**, **B**, **C**, **D**) for Unc13-C1^{HK} mutant flies. (**I** and **J**) Shown in Fig.1A before. (**I**) Representative example traces of spontaneous mEPSPs from wildype control (w1118) synapses (Unc13-C2B^{KW} experiment) before (grey, top) and after 1-minute octopamine incubation (blue, bottom) and quantification of mEPSP amplitudes. (**J**) Representative AP-evoked eEPSP responses (average of five repetitions in one cell) from wildtype control (w1118) synapses (Unc13-C2B^{KW} experiment) and quantification of eEPSP amplitudes before (grey) and after octopamine incubation (blue). (**K**, **N**) Comparison of the fold change in eEPSP amplitudes after 1 minute of octopamine incubation between Ctrl genotype (blue) and Unc13-C2B^{KW} mutants (green) or Unc13-C1^{HK} mutants (red). (**L, M**) Same analysis as in (**I**, **J**) for Unc13-C1^{HK} mutant flies. Number of cells (n) and animals (N) investigated: n/N: n/N(*Ctrl*, **A**) = 9/9, n/N(Unc13-C2B^{KW}, **B**) += 8/8, n/N(Ctrl, **D**) = 11/11, n/N(Unc13-C1^{HK}, **E**) = 11/11, n/N(Ctrl, G) = 9/9, n/N(Ctrl, **H**) = 10/10, n/N(Ctrl, **I**) = 11/11, n/N (Ctrl, **J**) = 12/12. For exact genotypes see methods. For details see table S14. Data depict mean values ± SEM. Difference between means was tested with paired parametric t-test (**B, C, I, J, K, L, F, G**) or Statistical analysis with unpaired, Mann-Whitney U test (**A, D, E, H**). n.s., p > 0.05; **p % 0.01; ***p % 0.001.

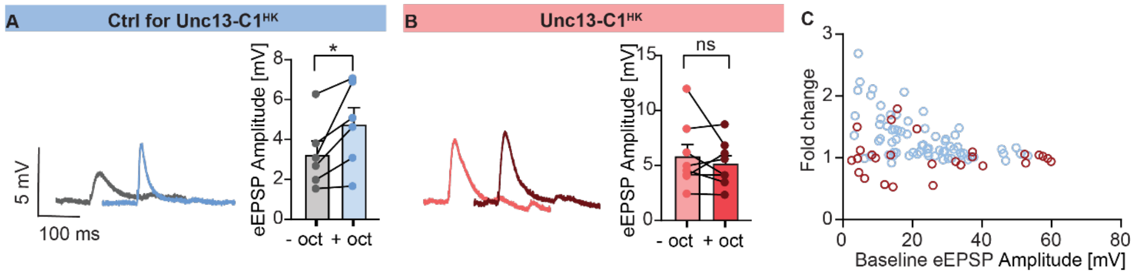

Figure S10: Octopamine incubation does not increase low baseline eEPSP amplitudes in Unc13-C1^{HK} mutants. (**A** and **B**) Representative AP-evoked eEPSP responses (average of five repetitions in one cell) at 0.1mM Ca^{2+} before octopamine (-oct, grey/light red) and after 1-minute incubation with 20 µM octopamine (+oct, blue/dark red) and quantification of eEPSP amplitudes. (**C**) Fold change of eEPSP amplitudes of control genotypes and Unc13-C1^{HK} at different Ca^{2+} concentrations (pooled experiments in the presence of 0.1, 0.3 and 0.4 mM external Ca^{2+}). Number of cells (n) and animals (N) investigated: n/N: n/N(*Ctrl*, **A**) =6/6, n/N(*Ctrl*, **C**)=70/70; n/N(*Unc13-C1^{HK}*, **A**) =8/8, n/N(*nc13-C1^{HK}*, **C**)=27/27. For exact genotypes see methods. Data depict mean values ± SEM. Difference between means was tested with paired parametric t-test). For details see table S15. n.s., p > 0.05; **p % 0.01; ***p % 0.001.

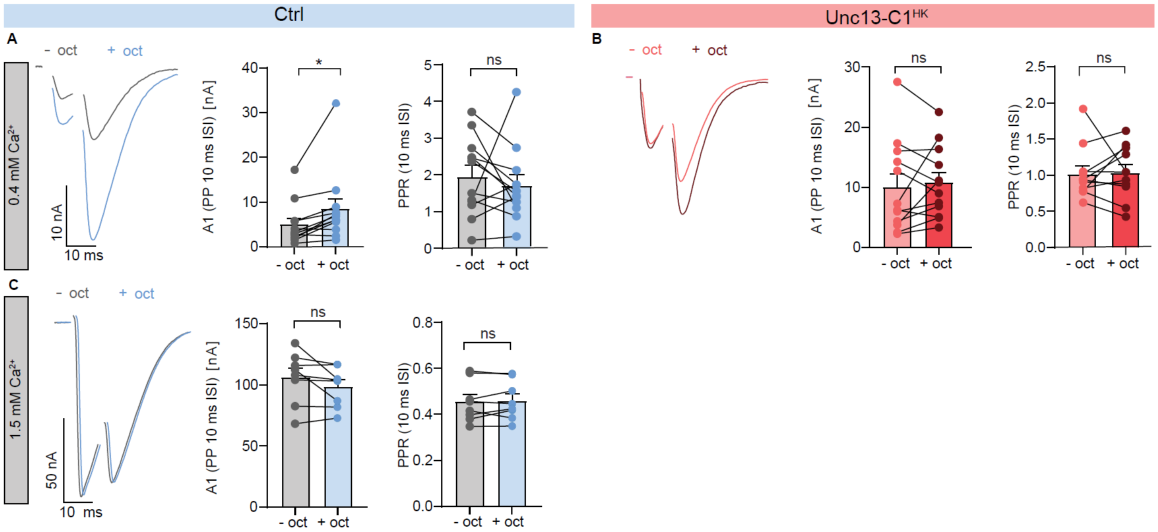

Figure S11: Octopamine acutely potentiates neurotransmitter release in control synapses at 0.4 mM, but not 1.5 mM external Ca^{2+} and potentiation is blocked in the Unc13-C1^{HK} mutant at 0.4 mM. (**A**) Analysis of wildtype control (Ctrl, w1118) animals at 0.4 mM extracellular Ca^{2+} concentration. Left: example traces of AP-evoked paired-pulse responses before 1-minute octopamine incubation (−oct, grey) or after 1-minute 20 µM octopamine incubation (+oct, blue). Middle: quantification of $eEPSC_1$ amplitudes before and after octopamine. Right: quantification of PPR ratios (10 ms interstimulus interval [ISI]). (**B**) same as (**A**) but in the Unc13-C1^{HK} mutant. (**C**) Same as (**A**) at 1.5 mM extracellular Ca^{2+} concentration. Number of cells (n) and animals (N) investigated: n/N: n/N(*Ctrl*, **A**) = 12/12, (**C**) = 8/8, n/N(Unc13-C1^{HK}, **B**) = 12/12., For exact genotypes see methods. Data depict mean values ± SEM. Statistical analysis with paired parametric t-test. For details see table S16. n.s., p > 0.05; *p % 0.05; **p % 0.01; ***p % 0.001

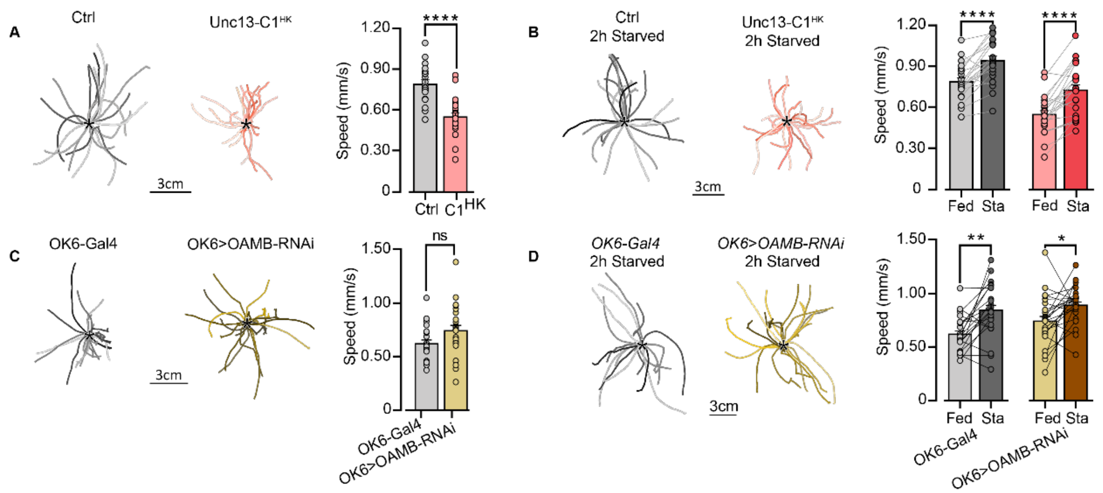

Figure S12: Unc13-C1^{HK} mutants and motoneuron OAMB KD exhibit distinct larval locomotor responses but preserve starvation-induced crawling adaptation. (**A**) 1-minute trajectories of third-instar larvae crawling filmed at 15 frames/s (*, starting position) from wildtype (w1118) larvae (Ctrl, grey traces) and from Unc13-C1^{HK} mutant larvae (C1^{HK}, light red traces). In comparison to control larvae, crawling speed is diminished in Unc13-C1^{HK} mutants. (**B**) Same fly larvae as in A were starved over a two-hour period and trajectories observed again for 1 minute. (**C**) Crawling speed was not affected by motoneuron OAMB KD (light brown traces, *OK6-Gal4>UAS-OAMB-RNAi*) compared to the control genotype (grey traces, OK6-Gal4); light brown traces, *OK6-Gal4>UAS-OAMB-RNAi*). (**D**) 2h-starvation significantly increases crawling speed in both groups. Number of animals (N) investigated: N(*Ctrl*, **A, B**)=22; N(*C1^{HK}*, **A, B**)=22; N(*OK6-Gal4*, **C, D**)=21; N(*OK6-Gal4>UAS-OAMB-RNAi*, **C, D**)=23. Statistical analysis with unpaired (**A,C**) or paired parametric t-test (**B,D**). Data depict mean values ± SEM. For details see table S18. P>0.05; ns, * p < 0.05; **p < 0.01; ***p < 0.001.

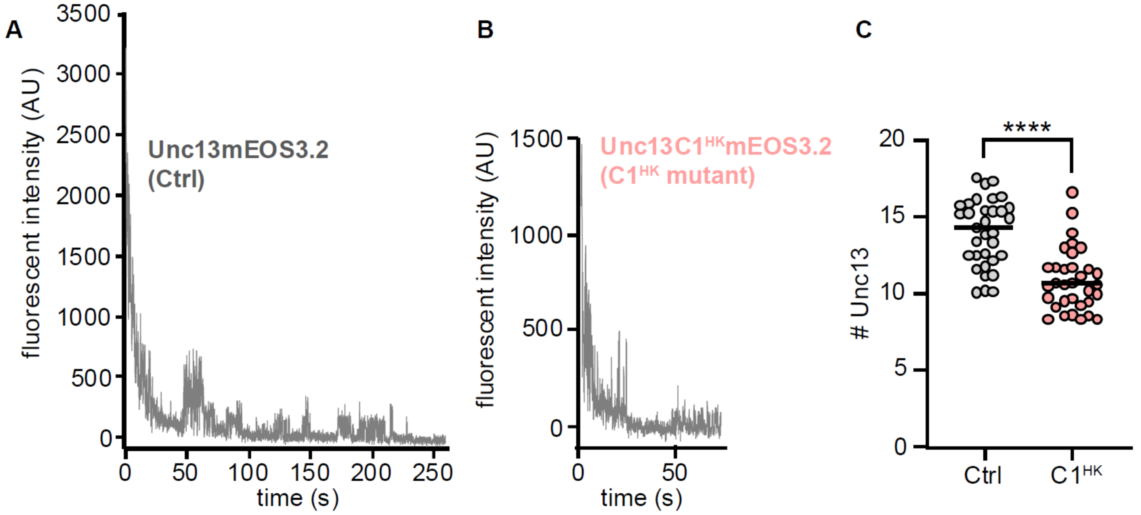

Figure S13: Molecular counting of Unc13-C1^{HK}-mEOS3.2 mutant sptPALM live imaging experiments in *Drosophila* larvae. Estimation of relative channel numbers based on the blinking fluorescent emission of mEOS3.2 tagged UNC13 molecules via bleach curve analysis. The average fluorescent intensity of individual synapses was recorded for 250s and 50s. To estimate signal bleach-steps, fluorescent intensity measurements over time were normalized by first subtracting all intensity values to the average background signal and second to the average amplitude of single fluorophore events at the end of the recording. Bleach curve fluorescent intensity measurements of Unc13mEOS3.2 controls (Ctrl) (**A**), and (**B**) Unc13-C1^{HK}-mEOS3.2 mutant (C1^{HK}). For comparison (**C**), quantification of relative Unc13 numbers extracted from a subset of the same data analyzed in of the live sptPALM experiments of Fig. 5B-K. Number of NMJs (n) and animals (N) and number of AZs (X) investigated: n/N/X(*Ctrl in C*) = *3*/2/33; n/N/X(C1^{HK}) = *5*/2/33. Data distribution was statistically tested with an unpaired T-test. For details see table S20. Statistical significance is denoted as asterisks: *$p < 0.01$,***$p < 0.001$, and ****$p < 0.0001$.

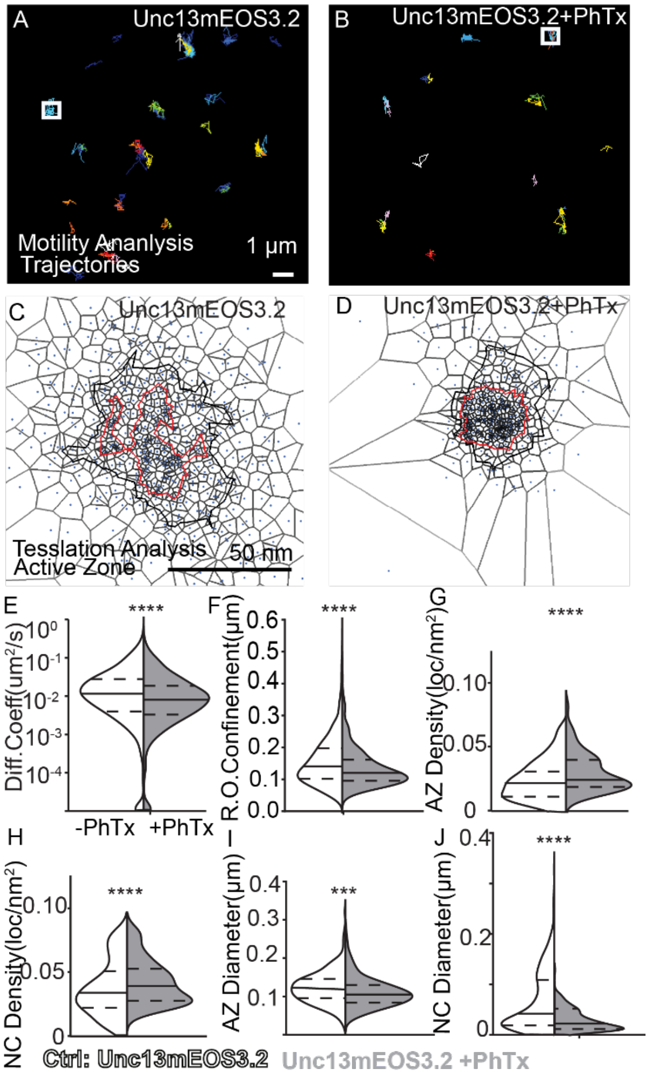

Figure S14: Live *in vivo* imaging of endogenously tagged Unc13 molecules reveals slowed diffusion and nanoscale compaction upon 20-minute PhTx treatment. Live sptPALM imaging of endogenously tagged Unc13mEOS3.2 at muscle 4 NMJs, performed in 0.4 mM Ca^{2+} and 10 mM Mg^{2+} containing HL3.1, before (Internal control/Ctrl) and after incubation with 50 µM Philanthotoxin (PhTx) in HL3.1 imaging buffer. (**A-D**) Images show representative sptPALM recordings depicting trajectory maps of boutons (**A-B**), and tessellation analysis representations (**C-D**) of Unc13mEOS3.2 AZs without and with a 10-min PhTx incubation and imaging after 20-minute after treatment. (**E** and **F**) Quantification of diffusion coefficients and radii of confinement from live Unc13 channel sptPALM imaging. (**G-J**) Tessellation analysis from the same sptPALM dataset was analyzed for diameters and densities of Unc13 localizations within AZ (G-H) and NC cluster (**I-J**) boundaries. Here, 7-16 animals per condition generated 175 AZs (control), 95 AZs (PhTx), respectively, that were analyzed. Number of NMJs (n), animals (N), number of AZs (X) and number of individual trajectories (Y) investigated: n/N/X/Y(Ctrl) = 46/7/276/5736; n/N/X/Y (PhTx) = 35/7/228/2568; Data depict mean values ± SEM. For details see table S21. Statistical significance is denoted as asterisks: **p < 0.01, ***p < 0.001, and ****p < 0.0001. Data distribution was statistically tested with a Kolmogorov-Smirnov test. AU, arbitrary units. Scale bars, 1 µm (**A**) and 50 nm (**C**).

Supplementary tables

Table S1: Key resources. Table provides an overview of the resources employed in this study, including antibodies, reagents, drugs, *Drosophila melanogaster* strains, consumables, software and algorithms and other resources important for this study.

**Antibodies**		
**Reagent or Resource**	**Source**	**Identifier**
Mouse-NC82 monoclonal (Wagh et al. 2012)	Developmental Studies Hybridoma Bank	Nc82 (Registry ID: AB_2314866)
Rabbit-Anti-GFP	Invitrogen	A11122 (Registry ID: AB_221569)
Anti-Rabbit Alexa 488	ThermoFisher	2284614 (Registry ID: AB_143165)
Anti-Mouse CY3	Jackson ImmunoResearch	115-167-003 (Registry ID: AB_2338709)
Anti-Goat HRP 647	Jackson ImmunoResearch	123-605-021 (Registry ID: AB_2338967)
Guinea Pig-Unc13A N-Term	Gift from Stefan Sigrist	
Rabbit-Unc13A N-Term	Gift from Stefan Sigrist	
Rabbit-Unc13A N-term	Absea Biotechnology GmbH	
Guinea Pig- Unc13 antibody	Absea Biotechnology GmbH	
Native Goat Serum	Sigma	037K7407V
Anti-Guinea 488	Invitrogen	2304259 (RRID: AB_2534117)
Alexa Fluor® 488 AffiniPure™ Goat Anti-Horseradish Peroxidase	Jackson ImmunoResearch	123-545-021 (RRID: AB_2338965)
**Reagents**		
**Reagent or Resource**	**Source**	**Identifier**
4% Paraformaldehyde in PBS	HistoLab	HL96753.1000
Triton-X100	Sigma Aldrich	X100
D-(+)-Trehalose	Sigma Aldrich	SLCF8650
HEPES	Sigma Aldrich	H3375
Magnesium Chloride Hexahydrate	Sigma Aldrich	M9272
Phosphate Buffered Solution	Gibco	18912-014
Potassium Chloride	Supelco	1.04933.0500
Sodium Bicarbonate	Sigma Aldrich	S8875
Sodium Chloride	Sigma Aldrich	57663
Sucrose	Millipore	1.07687.1000
**Drugs**		
**Reagent or Resource**	**Source**	**Identifier**
DMSO	Sigma	276855
Octopamine	Sigma	O0250
PMA	Sigma	P1585

PLC control drug (U73343)	Tocris	4133	
PLC inhibitor (U73122)	Tocris	1268	
**Experimental models**			
**Organisms/strains**	**Strain**	**FlyBase ID**	**Reference**
D.melanogaster	Wildtype: w1118	FBal0018186	Bloomington / WellGenetics
D.melanogaster	OK6-Gal4/II	FBti0023258	Aberle et al. 2002
D.melanogaster	UAS-OAMB-RNAi/III	FBst0031171	Perkins et al. 2015
D.melanogaster	UAS-Syt-mScarlet-GCaMP8f/III	FBti0219286	Chen et al. 2024
D.melanogaster	UAS-PLC-RNAi/II	FBgn0004611	Shortridge et al. 1991
D.melanogaster	UAS-Unc13A-RNAi/III	FBtp0131529	Reddy-Alla et al. 2017
D.melanogaster	UAS-Δ-N-term-Unc13/III	FBal0344167	Reddy-Alla et al. 2017
D.melanogaster	Unc13-C2B^{KW}		This paper
D.melanogaster	Unc13-C1^{HK}		This paper
D.melanogaster	Unc13mEOS3.2-C1^{HK}		This paper
D.melanogaster	Unc13mEOS3.2		This paper
**Consumables**			
**Reagent or Resource**	**Source**	**Identifier**	
Glass Slides	Carl Roth	H868.1	
Cover Slips #1.5	VWR	631-0147	
VectaShield	Vector Labs	H-1000-10	
**Software and algorithms**			
**Resource**	**Source**	**Identifier**	
Fiji	NIH (1)	https://fiji.sc/	
GraphPad Prism	GraphPad Software	version 10.2.3	
ImageJ	NIH	Version: v1.54f	
Imspector (16.3.14287-w2129)	Abberior Instruments	https://imspector.abberior-instruments.com/	
Microsoft Office Excel, Word, and PowerPoint 2010	Microsoft	www.office.com/?omkt=de-de	
MetaMorph Microscopy Automation and Image Analysis Software incl. PALM tracer built-in application	Molecular Devices (2)	www.moleculardevices.com/systems/metamorph-research-imaging/metamorph-microscopy-automation-and-image-analysis-software	
NanoJ-SRRF (ImageJ/Fiji plugin)	(3)	http://sites.imagej.net/NanoJ/; http://sites.imagej.net/NanoJ-SRRF/	

SR-Tesseler	(4)	https://github.com/flevet/SR-Tesseler/releases/tag/v1.0
Python pyABF package for Python 3.10 (Harden, 2022); pyABF 2.3.5	Python 3.10	https://pypi.org/project/pyabf
ThunderSTORM extracted PALM localization maps	(5)	https://zitmen.github.io/thunderstorm/
Adobe illustrator		2024 v28.71
Clampfit software	Molecular Devices	11.2.2.17
R Core Team (2024)	R version 4.4.2	https://www.R-project.org/
**Other**		
**Reagent or Resource**	**Source**	**Identifier**
Aberrior Infinity Line Confocal and 2D STED super-resolution microscope	Aberrior (Max Planck Innovation, Germany)	
Olympus IX83 Inverted Microscope with 2 decks microscope Frame	Olympus (Evident Europe, Germany)	IX83 two deck system, https://www.olympus-lifescience.com/en/microscopes/inverted/ix83/
sptPALM Microscope (Nikon Eclipse Ti) with EMCCD camera (iXon+ 897, Andor Technology) controlled by NIS-Elements (Nikon	Nikon and Andor Technology	https://www.microscope.healthcare.nikon.com/en_EU/products/inverted-microscopes/eclipse-ti-series<br><br>https://andor.oxinst.com/products/emccd-cameras?ppc_keyword=emccd&gad_source=1&gclid=Cj0KCQiA57G5BhDUARIsACgCYnw3QNbXzOjZEQRUekLbssWCQ9G75F5LSUuJVQb6VhgQ9kJXGgYDWhAaApmYEALw_wcB
PiVR Hardware		https://pivr.readthedocs.io/en/latest/

Table S2: Statistical information for experimental data from Figure 1. This table provides comprehensive statistical summaries for the experimental data illustrated in Figure 1. It includes details on the tested variables, experimental groups, mean ± SEM values, the specific statistical tests employed, significance levels, and the total number of cells and animals analyzed.

Figure 1

	Measure	Group	Mean	SEM	Test Type	Significance level	n (cells)	N (animals)
A	mEPSP amplitude [mV]	ctrl (w1118) -oct	1.105	0.155	paired parametric t-test	0.0711	12	12
		ctrl (w1118) +oct	1.003	0.141			12	12
	eEPSP amplitude [mV]	ctrl (w1118) -oct	18.77	2.886	paired parametric t-test	0.001	12	12
		ctrl (w1118) +oct	24.37	2.893			12	12
B	Foldchange	w1118/xw1118					70	70
C	eEPSP amplitude [mV]	UAS-OAMB-RNAi x Ok6 -oct	8.766	4.152	paired parametric t-test	0.6552	11	11
		UAS-OAMB-RNAi x Ok6 +oct	8.548	4.332			11	11
D	eEPSP amplitude [mV]	ctrl (w1118)PLC blocker; -oct	22.59	2.642	paired parametric t-test	0.6814	12	12
		ctrl (w1118) PLC blocker; +oct	22.17	2.643			12	12
E	eEPSP amplitude [mV]	PLC-RNAi/Ok6; -oct	34.55	4.591	paired parametric t-test	0.04	8	8
		PLC-RNAi/Ok6; +oct	37.76	4.257			8	8
F	Foldchange	before	1	0	Friedman test		15	15
		1min	1.316	0.1825		0.0443	15	15
		10min	1.275	0.1563		0.6634	15	15
		20min	1.417	0.1726		0.0187	15	15
		30min	1.376	0.2418		0.7368	15	15
G	Mean BRP intensity (a.u.)	ctrl (w1118) DMSO	326.5	12.05	unpaired parametric t-test	0.0166	21	7
		ctrl (w1118) Oct	366	9.851			19	7
	Mean Unc13 intensity (a.u.)	ctrl (w1118) DMSO	154.2	7.355	unpaired parametric t-test	0.0044	21	7
		ctrl (w1118) Oct	183.7	6.266			19	7

Table S3: Statistical information for experimental data from Figure S1. This table provides comprehensive statistical summaries for the experimental data illustrated in Fig.S1. It includes details on the tested variables, experimental groups, mean ± SEM values, the specific statistical tests employed, significance levels, and the total number of cells and animals analyzed.

Figure S1

	Measure	Group	Mean	SEM	Test Type	Significance level	n (cells)	N (animals)
A	eEPSP amplitude [mV]	ctrl (w1118) -oct	8.308	1.222	paired parametric t-test	0.0071	6	6
		ctrl (w1118) +oct	12.88	1.638			6	6
B	eEPSP amplitude [mV]	ctrl (w1118) -DMSO	5.95	0.9045	paired parametric t-test	0.1177	5	5
		ctrl (w1118) +DMSO	7.018	1.093			5	5
C	Frequency [Hz]	Ctrl (w1118) -oct	3.658	0.3535	paired parametric t-test	0.2217	12	12
		Ctrl (w1118) +oct	3.375	0.3979			12	12
D	peak (ΔF-F)/F0 [unitless]	Syt::GCaMP8f - 1AP (light gray)	0.0615	0.009	paired parametric t-test	0.6476	8	8
		Syt::GCaMP8f - 1AP (dark gray)	0.0548	0.0165			8	8
	peak (ΔF-F)/F0 [unitless]	Syt::GCaMP8f - oct	0.0591	0.00911	paired parametric t-test	0.6418	10	10
		Syt::GCaMP8f +oct	0.0562	0.00812			10	10
E	peak (ΔF-F)/F0 [unitless]	Syt::GCaMP8f - 20AP (light gray)	0.1228	0.0258	paired parametric t-test	0.408	6	6
		Syt::GCaMP8f - 20AP (dark gray)	0.1101	0.0251			6	6
	peak (ΔF-F)/F0 [unitless]	Syt::GCaMP8f - oct	0.168	0.0543	paired parametric t-test	0.2704	8	8
		Syt::GCaMP8f +oct	0.201	0.0539			8	8
F	eEPSP amplitude [mV] (Baseline)	UAS-OAMB-RNAi x W1118 - oct	10.07	2.413	Mann-Whitney U test	0.9408	9	9
		UAS-OAMB-RNAi x Ok6-Gal4 -oct	8.766	1.252			11	11
G	eEPSP amplitude [mV]	UAS-OAMB-RNAi x Ok6-Gal4 -oct	10.07	2.413	paired parametric t-test	0.0151	9	9
		UAS-OAMB-RNAi x Ok6-Gal4 +oct	12.54	2.51			9	9
H	Fold change	UAS-OAMB-RNAi x W1118	1.424	0.1586	Mann-Whitney U test	0.02	9	9
		UAS-OAMB-RNAi x Ok6-Gal4 oct	0.9928	0.06493			11	11

Table S4: Statistical information for experimental data from Figure S2. This table provides comprehensive statistical summaries for the experimental data illustrated in Fig.S2. It includes details on the tested variables, experimental groups, mean ± SEM values, the specific statistical tests employed, significance levels, and the total number of cells and animals analyzed.

Figure S2

	Measure	Group	Mean	SEM	Test Type	Significance level	n (cells)	N (animals)
B	mEPSP amplitude [mV]	ctrl (w1118) ctrl drug -oct	0.9697	0.0469	paired parametric t-test	0.9899	13	13
		ctrl (w1118) ctrl drug **+oct**	0.9701	0.03834			13	13
	eEPSP amplitude [mV]	ctrl (w1118) ctrl drug -oct	19.91	3.055	paired parametric t-test	0.0005	13	13
		ctrl (w1118)ctrl drug **+oct**	24.64	2.943			13	13
C	mEPSP amplitude [mV]	ctrl (w1118) PLC blocker; -oct	1.243	0.15	paired parametric t-test	0.13	12	12
		ctrl (w1118) PLC blocker; +oct	1.113	0.1007			12	12
	eEPSP amplitude [mV]	ctrl (w1118)PLC blocker; -oct	22.59	2.642	paired parametric t-test	0.6814	12	12
		ctrl (w1118) PLC blocker; +oct	22.17	2.643			12	12
D	Fold change	ctrl (w1118) ctrl drug	1.395	0.1359	Mann-Whitney U test	0.001	13	13
		ctrl (w1118) PLC blocker	0.9843	0.04622			12	12
F	mEPSP amplitude [mV]	PLC-RNAi/+ -oct	0.9816	0.09479	paired parametric t-test	0.6047	8	8
		PLC-RNAi/+ **+oct**	0.9554	0.1015			8	8
	eEPSP amplitude [mV]	PLC-RNAi/+ -oct	24.99	4.967	paired parametric t-test	0.0013	8	8
		PLC-RNAi/+ **+oct**	33.03	4.894			8	8
G	mEPSP amplitude [mV]	PLC-RNAi/Ok6; -oct	1.37	0.1352	paired parametric t-test	0.6417	8	8
		PLC-RNAi/Ok6; +oct	1.304	0.2228			8	8
	eEPSP amplitude [mV]	PLC-RNAi/Ok6; -oct	34.55	4.591	paired parametric t-test	0.04	8	8
		PLC-RNAi/Ok6; +oct	37.76	4.257			8	8
H	Fold change	PLC-RNAi/+	1.424	0.09228	Mann-Whitney U test	0.0148	8	8
		PLC-RNAi/Ok6	1.122	0.04868			8	8
I	mEPSP amplitude [mV]	(w1118) ctrl drug	0.9697	0.0469	Mann-Whitney U test	0.0976	13	13
		(w1118) PLC Blocker	1.243	0.15			12	12
	eEPSP amplitude [mV]	(w1118) ctrl drug	19.91	3.055	Mann-Whitney U test	0.5033	13	13
		(w1118) PLC Blocker	22.59	2.642			12	12
J	Frequency [Hz]	ctrl (w1118) ctrl drug -oct	3.862	0.2635	paired parametric t-test	0.6487	13	13
		ctrl (w1118) ctrl drug +oct	3.749	0.2083			13	13
	Frequency [Hz]	ctrl (w1118) PLC blocker; -oct	3.167	0.2338	paired parametric t-test	0.2828	12	12
		ctrl (w1118) PLC blocker; +oct	2.992	0.2323			12	12
K	Fold change (Frequency)	ctrl (w1118) ctrl drug	1.009	0.07314	Mann-Whitney U test	0.9362	13	13
		ctrl (w1118) PLC blocker	0.958	0.05315			12	12
L	mEPSP amplitude [mV]	UAS-PLC-RNAixw1118	0.9816	0.09479	Mann-Whitney U test	0.0362	8	8
		UAS-PLC-RNAixok6-Gal4	1.37	0.1352			8	8
	eEPSP amplitude [mV]	UAS-PLC-RNAixw1118	24.99	4.967	Mann-Whitney U test	0.1304	8	8
		UAS-PLC-RNAixok6-Gal4	34.55	4.591			8	8
M	Frequency [Hz]	UAS-PLC-RNAixw1118 -oct	2.125	0.2922	paired parametric t-test	0.4636	8	8
		UAS-PLC-RNAixw1118 +oct	2.321	0.3608			8	8
	Frequency [Hz]	UAS-PLC-RNAixok6-Gal4 -oct	2.979	0.4428	paired parametric t-test	0.32	8	8
		UAS-PLC-RNAixok6-Gal4 +oct	2.625	0.2961			8	8
N	Fold change (Frequency)	UAS-PLC-RNAixw1118	1.122	0.1216	Mann-Whitney U test	0.3671	8	8
		UAS-PLC-RNAixok6-Gal4	0.9426	0.08128			8	8

Table S5: Statistical information for experimental data from Figure S3. This table provides comprehensive statistical summaries for the experimental data illustrated in Fig.S3. It includes details on the tested variables, experimental groups, mean ± SEM values, the specific statistical tests employed, significance levels, and the total number of cells and animals analyzed.

Figure S3

	Measure	Group	Mean	SEM	Test Type	Significance level	n (cells)	N (animals)
A	Mean BRP intensity (a.u.)	1 minute DMSO	170	10.81	Unpaired Parametric t-Test	0.0503	18	6
		1 minute Octopamine	198	7.869			15	5
		30 minute DMSO	158.4	12.29	Unpaired Parametric t-Test	0.041	18	6
		30 minute Octopamine	189.9	8.315			18	6
	Mean Unc13 intensity (a.u.)	1 minute DMSO	161	5.275	Unpaired Parametric t-Test	0.0049	18	6
		1 minute Octopamine	184.7	5.817			15	5
		30 minute DMSO	159.5	4.279	Unpaired Parametric t-Test		18	6
		30 minute Octopamine	174.7	2.717		0.005	18	6

Table S6: Statistical information for experimental data from Figure 2. This table provides comprehensive statistical summaries for the experimental data illustrated in Fig.2. It includes details on the tested variables, experimental groups, median ± SEM values, the specific statistical tests employed, significance levels, the total number of cells, animals, active zones and trajectories analyzed.

Figure 2

	Measure	Group	Median	SEM	Test Type	Statistical test value	P-value	Significance	n (cells)	N (animals)	Azs	Trajectories
L	Median transformed diffusion coefficient (Log 10) ($\mu m^2/s$)	Unc13mEOS-IntCtrl	-1.81	0.01942	Kolmogorov-Smirnov D	0.2186	<0.0001	****	7	7		2052
		Unc13mEOS-1MinOct	-2.13	0.01588					7	7		2505
M	Median ROC (µm)	Unc13mEOS-IntCtrl	0.1632	0.001441	Kolmogorov-Smirnov D	0.2668	<0.0001	****	7	7		2052
		Unc13mEOS-1MinOct	0.1159	0.001157					7	7		2505
N	Median Azdiameter (nm)	Unc13mEOS-IntCtrl	70.48	3.223	Kolmogorov-Smirnov D	0.4088	<0.0001	****	7	7	106	
		Unc13mEOS-1MinOct	52.27	1.896					7	7	86	
P	Median Nc diameter (nm)	Unc13mEOS-IntCtrl	37.11	1.59	Kolmogorov-Smirnov D	0.3133	0.0002	***	7	7	106	
		Unc13mEOS-1MinOct	27.49	0.9408					7	7	86	
O	Median AZ Density ($locs/nm^2$)	Unc13mEOS-IntCtrl	0.07083	0.01197	Kolmogorov-Smirnov D	0.311	0.0003	***	7	7	106	
		Unc13mEOS-1MinOct	0.1111	0.02665					7	7	86	
Q	Median NC Density (locs/nm2)	Unc13mEOS-IntCtrl	0.1337	0.02233	Kolmogorov-Smirnov D	0.3208	0.0001	***	7	7	106	
		Unc13mEOS-1MinOct	0.3255	0.04929					7	7	86	

Table S7: Statistical information for experimental data from Figure S4. This table provides comprehensive statistical summaries for the experimental data illustrated in Fig.S4. It includes details on the tested variables, experimental groups, mean ± SEM values, the specific statistical tests employed, significance levels, and the total number of cells and animals analyzed.

Figure S4

	Measure	Group	Mean	SEM	Test Type	Significance level	n (cells)	N (animals)
A	mEPSP amplitude [mV]	Ctrl (W1118)	0.5145	0.0516	Unpaired Parametric t-Test	0.1132	13	13
		Unc13mEOS3.2	0.6252	0.04327			13	13
B	eEPSP amplitude [mV]	Ctrl (W1118)	42.99	3.085	Unpaired Parametric t-Test	0.2705	13	13
		Unc13mEOS3.2	38.82	2.046			13	13
C	Mean BRP intensity (a.u.)	ctrl (w1118)	220.4	3.868	unpaired parametric t-test	0.2087	12	4
		Unc13mEOS3.2	226.7	3.065			18	6
	Mean Unc13 intensity (a.u.)	ctrl (w1118)	199.1	3.982	unpaired parametric t-test	0.40395	12	4
		Unc13mEOS3.2	198.1	11.76			18	6

Table S8: Statistical information for experimental data from Figure S5. This table provides comprehensive statistical summaries for the experimental data illustrated in Fig.S5. It includes details on the tested variables, experimental groups, median ± SEM values, the specific statistical tests employed, significance levels, the total number of cells, animals, active zones and trajectories analyzed.

Figure S5

	Measure	Group	Median	SEM	Test Type	Statistical test value	P-value	Significance	n (cells)	N (animals)	Azs	Trajectories
G	Median transformed diffusion coefficient (Log 10) ($\mu m^2/s$)	Unc13mEOS-IntCtrl	-1.876	0.01998	Kolmogorov-Smirnov D	0.8974	0.8974	n.s.	10	10		1437
		Unc13mEOS-1MinDMSO	-1.86	0.01323					10	10		2966
H	Median ROC (µm)	Unc13mEOS-IntCtrl	0.1485	0.003207	Kolmogorov-Smirnov D	0.7876	0.7876	n.s.	10	10		1437
		Unc13mEOS-1MinDMSO	0.1476	0.001519					10	10		2966
I	Median Azdiameter (µm)	Unc13mEOS-IntCtrl	68.48	1.543	Mann Whitney test	0.4347	0.9291	n.s.	10	10	214	
		Unc13mEOS-1MinDMSO	69.55	1.468					10	10	201	
K	Median Nc diameter (µm)	Unc13mEOS-IntCtrl	30.09	1.17	Mann Whitney test	0.9464	0.4347	n.s.	10	10	214	
		Unc13mEOS-1MinDMSO	30.14	1.355					10	10	201	
J	Median AZ Density ($locs/nm^2$)	Unc13mEOS-IntCtrl	0.06717	0.001766	Mann Whitney test	0.8064	0.0513	n.s.	10	10	214	
		Unc13mEOS-1MinDMSO	0.07045	0.001601					10	10	201	
L	Median NC Density (locs/nm2)	Unc13mEOS-IntCtrl	0.1468	0.003452	Mann Whitney test	0.5574	0.6005	n.s.	10	10	214	
		Unc13mEOS-1MinDMSO	0.15	0.004256					10	10	201	

Table S9: Statistical information for experimental data from Figure S6. This table provides comprehensive statistical summaries for the experimental data illustrated in Fig.S6. It includes details on the tested variables, experimental groups, the specific statistical tests employed, significance levels, and the total number of cells and animals analyzed.

Figure S6

	Measure	Group	Mean	SEM	Test Type	Significance level	n (cells)	N (animals)
C	Bleach Curve Analysis	Unc13mEOS internal Control	10.7	0.377	t-test (unpaired)	0.2061	24	3
		Unc13mEOS + oct	11.76	0.5845			24	3

Table S10: Statistical information for experimental data from Figure S7. This table provides comprehensive statistical summaries for the experimental data illustrated in Fig.S7. It includes details on the tested variables, experimental groups, median ± SEM values, the specific statistical tests employed, significance levels, the total number of cells, animals, active zones and trajectories analyzed.

Fig S7

	Measure	Group	Median	SEM	Test Type	Statistical test value	P-Value	Significance	n (cells)	N (animals)	Azs	Trajectories
E	Median transformed diffusion coefficient (Log 10) ($\mu m^2/s$)	Unc13mEOS	-1.804	0.01441	Kolmogrov smirnof -test	0.3442	0.3442		8	8		2380
		Unc13mEOS +PMA	-1.789	0.01316				ns	8	8		2931
F	Median ROC (µm)	Unc13mEOS	0.1578	0.001745	Kolmogrov smirnof -test	0.0691	0.0691		8	8		2380
		Unc13mEOS +PMA	0.1545	0.001486				ns	8	8		2931
H	Median Azdiameter (nm)	Unc13mEOS	91.69	1.775	Kolmogrov smirnof -test	0.3922	<0.0001		8	8	251	
		Unc13mEOS +PMA	71.47	1.797				****	8	8	101	
J	Median Nc diameter (nm)	Unc13mEOS	16.21	0.9346	Kolmogrov smirnof -test	0.2183	<0.0001		8	8	251	
		Unc13mEOS +PMA	12.5	1.038				****	8	8	101	
G	Median AZ Density ($locs/nm^2$)	Unc13mEOS	0.0243	0.000529	Kolmogrov smirnof -test	0.7752	<0.0001		8	8	251	
		Unc13mEOS +PMA	0.04788	0.001873				****	8	8	101	
I	Median NC Density (locs/nm2)	Unc13mEOS	0.07717	0.002058	Kolmogrov smirnof -test	0.5331	<0.0001		8	8	251	
		Unc13mEOS +PMA	0.1516	0.004779				****	8	8	101	
Q	Median transformed diffusion coefficient (Log 10) ($\mu m^2/s$)	Unc13mEOS	-1.885	0.01019	Kolmogrov smirnof -test	0.1278	<0.0001		18	18		5924
		Unc13mEOS +10 min Oct	-2.066	0.008328				****	17	17		8747
R	Median ROC (µm)	Unc13mEOS	0.1477	0.0009566	Kolmogrov smirnof -test	0.18	<0.0001		18	18		5924
		Unc13mEOS +10 min Oct	0.1202	0.0008451				****	17	17		8747
S	Median Azdiameter (nm)	Unc13mEOS	76.64	1.974	Kolmogrov smirnof -test	0.1938	<0.0001		18	18	287	
		Unc13mEOS +10 min Oct	70.63	1.121				****	17	17	403	
U	Median Nc diameter (nm)	Unc13mEOS	36.11	1.412	Kolmogrov smirnof -test	0.1159	0.0297		18	18	287	
		Unc13mEOS +10 min Oct	30.39	0.9255				*	17	17	403	
T	Median AZ Density ($locs/nm^2$)	Unc13mEOS	0.04348	0.001672	Kolmogrov smirnof -test	0.3681	<0.0001		18	18	287	
		Unc13mEOS +10 min Oct	0.06577	0.002784				****	17	17	403	
V	Median NC Density (locs/nm2)	Unc13mEOS	0.1073	0.003484	Kolmogrov smirnof -test	0.3641	<0.0001		18	18	287	
		Unc13mEOS +10 min Oct	0.147	0.004709				****	17	17	403	

Table S11: Statistical information for experimental data from Figure 3. This table provides comprehensive statistical summaries for the experimental data illustrated in Fig.3. It includes details on the tested variables, experimental groups, mean ± SEM values, the specific statistical tests employed, significance levels, and the total number of cells and animals analyzed.

Figure 3

	Measure	Group	Mean	SEM	Test Type	Significance level	n (cells)	N (animals)
B	eEPSP amplitude [mV]	UAS-Unc13-RNAi x w1118 -oct	34.79	4.541	paired parametric t-test	0.0402	9	9
		UAS-Unc13-RNAi x w1118 +oct	37.36	4.561			9	9
C	eEPSP amplitude [mV]	UAS-Unc13-RNAi x OK6-Gal4; - oct	12.05	2.935	paired parametric t-test	0.2998	8	8
		UAS-Unc13-RNAi x OK6-Gal4, +oct	10.82	2.366			8	8
E	DMSO)fluoresence ratio	OK6-GAL4>UAS-Δ-N-term-Unc13	1.236	0.03802			9	3
	PMA fluoresence ratio	OK6-GAL4>UAS-Δ-N-term-Unc13	1.754	0.1046	Kruskal-Wallis test	0.025	8	3
	Octopamine fluoresence ratio	OK6-GAL4>UAS-Δ-N-term-Unc13	1.848	0.21		0.0054	9	3
F	DMSO fluoresence ratio	OK6-GAL4>UAS-Δ-N-term-Unc13, UAS-OAMB-RNAi	1.204	0.01346	Mann-Whitney U test	0.0516	23	8
	Octopamine fluoresence ratio	OK6-GAL4>UAS-Δ-N-term-Unc13, UAS-OAMB-RNAi	1.259	0.03915			22	8

Table S12: Statistical information for experimental data from Figure S8. This table provides comprehensive statistical summaries for the experimental data illustrated in Fig. S8. It includes details on the tested variables, experimental groups, mean ± SEM values, the specific statistical tests employed, significance levels, and the total number of cells and animals analyzed.

Figure S8

	Measure	Group	Mean	SEM	Test Type	Significance level	n (cells)	N (animals)
A	mEPSP amplitude [mV]	UAS-Unc13-RNAi x w1118 -oct	0.7669	0.07414	paired parametric t-test	0.7442	9	9
		UAS-Unc13-RNAi x w1118 +oct	0.7499	0.05981			9	9
B	mEPSP amplitude [mV]	UAS-Unc13-RNAi x w1118	0.7669	0.07414	Mann-Whitney U test	0.0152	9	9
		UAS-Unc13-RNAi x OK6-Gal4	1.212	0.1413			8	8
	eEPSP amplitude [mV]	UAS-Unc13-RNAi x w1118)	34.79	4.541	Mann-Whitney U test	0.0016	9	9
		UAS-Unc13-RNAi x OK6-Gal4	12.05	2.935			8	8
C	mEPSP amplitude [mV]	UAS-Unc13-RNAi x OK6-Gal4; - oct	1.212	0.1413	paired parametric t-test	0.0807	8	8
		UAS-Unc13-RNAi x OK6-Gal4; + oct	1.109	0.1108			8	8
D	Frequency [Hz]	UAS-Unc13A-RNAi x W1118 - oct	2.807	0.2561	paired parametric t-test	0.5868	9	9
		UAS-Unc13A-RNAi x W1118 +oct	2.933	0.2872			9	9
E	Frequency [Hz]	UAS-Unc13A-RNAi x Ok6-Gal4 - oct	2.558	0.2269	paired parametric t-test	0.7175	8	8
		UAS-Unc13A-RNAi x Ok6-Gal4 +oct	2.438	0.2358			8	8
F	Fold change (Frequency)	UAS-Unc13A-RNAi x W1118 +oct	1.057	0.09185	Mann-Whitney U test	0.4807	9	9
		UAS-Unc13A-RNAi x Ok6-Gal4 +oct	0.9799	0.09199			8	8
G	DMSO fluoresence ratio	OK6-GAL4>UAS-Δ-N-term-Unc13	1.243	0.02779	Mann-Whitney U test	0.000102	24	8
	oct fluoresence ratio	OK6-GAL4>UAS-Δ-N-term-Unc13	1.446	0.05379			21	7
H	DMSO fluoresence ratio	OK6-GAL4>UAS-Δ-N-term-Unc13	1.231	0.01551	Ordinary One-Way Anova		12	4
	PMA fluoresence ratio	OK6-GAL4>UAS-Δ-N-term-Unc13	1.621	0.09826		0.0018	11	4
	PhTx fluoresence ratio	OK6-GAL4>UAS-Δ-N-term-Unc13	1.559	0.09286		0.0081	11	4

Table S13: Statistical information for experimental data from Figure 4. This table provides comprehensive statistical summaries for the experimental data illustrated in Fig.4. It includes details on the tested variables, experimental groups, mean ± SEM values, the specific statistical tests employed, significance levels, and the total number of cells and animals analyzed.

Figure 4

	Measure	Group	Mean	SEM	Test Type	Significance level	n (cells)	N (animals)
B	mEPSP amplitude [mV]	Ctrl (W1118)	0.9522	0.1045	Mann Whitney test	0.9626	9	9
		C2B-KW	0.8744	0.06828			8	8
	eEPSP amplitude [mV]	Ctrl (W1118)	24.92	2.455	Mann Whitney test	0.0021	10	10
		C2B-KW	36.59	1.843			8	8
C	Mean BRP intensity (a.u.)	Ctrl (W1118)	275.1	14.31	Unpaired Parametric t-Test	0.8551	15	5
		C2B-KW	278.9	14.53			15	5
	Mean Unc13 intensity (a.u.)	Ctrl (W1118)	333	297.9	Unpaired Parametric t-Test	0.0067	15	5
		C2B-KW	297.9	8.609			15	5
E	mEPSP amplitude [mV]	Ctrl (W1118)	1.045	0.0853	Mann Whitney test	0.4491	12	12
		C1-HK	1.121	0.09234			11	11
	eEPSP amplitude [mV]	Ctrl (W1118)	19.9	3.246	Mann Whitney test	0.0129	12	12
		C1-HK	41.32	5.892			11	11
F	Mean BRP intensity (a.u.)	Ctrl (W1118)	304.6	10.63	Unpaired Parametric t-Test	0.3692	24	8
		C1-HK	290.3	11.38			24	8
	Mean Unc13 intensity (a.u.)	Ctrl (W1118)	356.6	5.4578	Unpaired Parametric t-Test	0.0437	24	8
		C1-HK	334.5	11.12			24	8
G	mEPSP amplitude [mV]	C2B-KW - oct	0.8744	0.06828	paired parametric t-test	0.4177	8	8
		C2B-KW +oct	0.894	0.07101			8	8
	eEPSP amplitude [mV]	C2B-KW - oct	36.59	1.843	paired parametric t-test	0.0469	8	8
		C2B-KW +oct	38.94	2.288			8	8
H	mEPSP amplitude [mV]	C1-HK - oct	1.121	0.09234	paired parametric t-test	0.362	11	11
		C1-HK +oct	1.171	0.09549			11	11
	eEPSP amplitude [mV]	C1-HK - oct	41.32	5.892	paired parametric t-test	0.9917	11	11
		C1-HK +oct	41.3	5.858			11	11

Table S14: Statistical information for experimental data from Figure S9. This table provides comprehensive statistical summaries for the experimental data illustrated in Fig.S9. It includes details on the tested variables, experimental groups, mean ± SEM values, the specific statistical tests employed, significance levels, and the total number of cells and animals analyzed.

Figure S9

	Measure	Group	Mean	SEM	Test Type	Significance level	n (cells)	N (animals)
A	Frequency (Hz)	Ctrl (W1118)	3.185	0.3169	Unpaired parametric t-test	0.2953	9	9
		C2B-KW	2.775	0.1822			8	8
B	Frequency (Hz)	Ctrl (W1118) -oct	3.185	0.3169	paired-parametric t-test	0.9904	9	9
		Ctrl (W1118) +oct	3.181	0.2247			9	9
C	Frequency (Hz)	C2B-KW -oct	2.775	0.1822	paired-parametric t-test	0.4727	8	8
		C2B-KW +oct	3	0.261			8	8
D	Fold Change	Ctrl (W1118)	1.045	0.09405	Mann Whithney test	0.8884	9	9
		C2B-KW	1.111	0.112			8	8
E	Frequency (Hz)	Ctrl (W1118)	3.558	0.3128	Mann Whithney test	0.2421	11	11
		C1-HK	2.912	0.3585			11	11
F	Frequency (Hz)	Ctrl (W1118) -oct	3.558	0.3128	paired-parametric t-test	0.6468	11	11
		Ctrl (W1118) +oct	3.482	0.3872			11	11
G	Frequency (Hz)	C1-HK -oct	2.912	0.3585	paired-parametric t-test	0.8845	11	11
		C1-HK +oct	2.942	0.2758			11	11
H	Fold Change	Ctrl (W1118)	0.9501	0.05829	Mann Whithney test	0.7969	11	11
		C1-HK	1.064	0.07922			11	11
I	mEPSP amplitude [mV]	ctrl (w1118)for C2B -oct	0.9522	0.1045	paired-parametric t-test	0.5374	9	9
		ctrl (w1118)for C2B +oct	1.009	0.08114			9	9
J	eEPSP amplitude [mV]	ctrl (w1118) for C2B-oct	24.92	2.455	paired-parametric t-test	0.0007	10	10
		ctrl (w1118) for C2B+oct	29.63	2.095			10	10
K	Fold change (eEPSP)	Ctrl (W1118)	1.237	0.065	Mann Whitney test	0.0343	10	10
		C2B-KW	1.063	0.0251			8	8
L	mEPSP amplitude [mV]	ctrl (w1118) C1-HK-oct	1.02	0.08336	paired-parametric t-test	0.5503	11	11
		ctrl (w1118)C1-HK +oct	0.9967	0.06775			11	11
M	eEPSP amplitude [mV]	ctrl (w1118)C1-HK -oct	19.9	3.246	paired-parametric t-test	0.0022	12	12
		ctrl (w1118)C1-HK +oct	25.75	3.623			12	12
N	Fold change (eEPSP)	Ctrl (W1118)	1.369	0.09969	Mann Whitney test	0.0017	12	12
		C1-HK	1.017	0.08955			11	11

Table S15: Statistical information for experimental data from Figure S10. This table provides comprehensive statistical summaries for the experimental data illustrated in Fig.S10. It includes details on the tested variables, experimental groups, mean ± SEM values, the specific statistical tests employed, significance levels, and the total number of cells and animals analyzed.

Figure S10

	Measure	Group	Mean	SEM	Test Type	Significance level	n (cells)	N (animals)
A	eEPSP amplitude [mV]	ctrl (w1118) -oct	3.231	0.692	paired parametric t-test	0.0336	6	6
		ctrl (w1118) +oct	4.741	0.8631			6	6
B	eEPSP amplitude [mV]	Unc13-C1HK -oct	5.841	1.068	paired parametric t-test	0.4113	8	8
		Unc13-C1HK +oct	5.17	0.7258			8	8
C	Foldchange	W1118/x; W1118					70	70
		Unc13-C1HK					27	27

Table S16: Statistical information for experimental data from Figure S11. This table provides comprehensive statistical summaries for the experimental data illustrated in Fig.S11. It includes details on the tested variables, experimental groups, mean ± SEM values, the specific statistical tests employed, significance levels, and the total number of cells and animals analyzed.

Figure S11

	Measure	Group	Mean	SEM	Test Type	Significance level	n (cells)	N (animals)
A	eEPSC amplitude [nA]	Ctrl (W1118) - Oct	4.95	1.36	paired paremetric t-test	0.0121	12	12
		Ctrl (W1118) + Oct	8.42	2.32			12	12
	PPR 10 ms ISI [nA]	Ctrl (W1118) - Oct	1.95	0.31	paired paremetric t-test	0.5332	12	12
		Ctrl (W1118) + Oct	1.71	0.29			12	12
C	eEPSC amplitude [nA]	Ctrl (W1118) - Oct	105.90	7.53	paired paremetric t-test	0.1341	8	8
		Ctrl (W1118) + Oct	98.33	5.69			8	8
	PPR 10 ms ISI [nA]	Ctrl (W1118) - Oct	0.45	0.03	paired paremetric t-test	0.5743	8	8
		Ctrl (W1118) + Oct	0.46	0.03			8	8
B	eEPSC amplitude [nA]	Unc13-C1(HK) - Oct	10.00	2.22	paired paremetric t-test	0.5901	12	12
		Unc13-C1(HK) + Oct	10.77	1.71			12	12
	PPR 10 ms ISI [nA]	Unc13-C1(HK) - Oct	1.02	0.11	paired paremetric t-test	0.8756	11	11
		Unc13-C1(HK) + Oct	1.04	0.11			11	11

Table S17: Statistical information for experimental data from Figure 5. This table provides comprehensive statistical summaries for the experimental data illustrated in Fig.5. It includes details on the tested variables, experimental groups, mean ± SEM values, the specific statistical tests employed, significance levels, and the total number of cells and animals analyzed.

Figure 5

	Measure	Group	Mean	SEM	Test Type	Significance level	n (cells)	N (animals)
B	eEPSC amplitude [nA], 0.4 mM Ca2+	Ctrl (W1118)	-5.87	1.04	Unpaired t-test	0.018008	12	6
		Unc13-C1(HK)	-13.07	2.62			12	6
	eEPSC amplitude [nA], 0.75 mM Ca2+	Ctrl (W1118)	-23.33	3.82	Unpaired t-test	0.011569	12	6
		Unc13-C1(HK)	-39.13	4.28			12	6
	eEPSC amplitude [nA], 1.5 mM Ca2+	Ctrl (W1118)	-71.07	3.81	Unpaired t-test	0.042431	12	6
		Unc13-C1(HK)	-85.57	5.55			12	6
	eEPSC amplitude [nA], 3 mM Ca2+	Ctrl (W1118)	-105.49	4.05	Unpaired t-test	0.947389	12	6
		Unc13-C1(HK)	-105.93	5.04			12	6
	eEPSC amplitude [nA], 6 mM Ca2+	Ctrl (W1118)	-122.94	4.07	Unpaired t-test	0.325488	12	6
		Unc13-C1(HK)	-115.21	6.52			12	6
C	PPR 10 ms ISI, 0.4 mM Ca2+	Ctrl (W1118)	1.87	0.23	Unpaired t-test	0.018812	12	6
		Unc13-C1(HK)	1.20	0.13			12	6
	PPR 10 ms ISI, 0.75 mM Ca2+	Ctrl (W1118)	1.69	0.25	Unpaired t-test	0.002073	12	6
		Unc13-C1(HK)	0..75	0.08			12	6
	PPR 10 ms ISI, 1.5 mM Ca2+	Ctrl (W1118)	0.69	0.05	Unpaired t-test	0.003607	12	6
		Unc13-C1(HK)	0.49	0.04			12	6
	PPR 10 ms ISI, 3 mM Ca2+	Ctrl (W1118)	0.58	0.02	Unpaired t-test	0.023396	12	6
		Unc13-C1(HK)	0.49	0.03			12	6
	PPR 10 ms ISI, 6 mM Ca2+	Ctrl (W1118)	0.64	0.02	Unpaired t-test	0.000188	12	6
		Unc13-C1(HK)	0.52	0.02			12	6
D	eEPSC amplitude [nA]	Ctrl (W1118) - PMA	3.84	0.86	Unpaired t-test	<0.0001	19	10
		Ctrl (W1118) + PMA	11.28	1.26			26	13
	PPR 10 ms ISI [nA]	Ctrl (W1118) - PMA	2.89	0.35	Unpaired t-test	<0.0001	17	9
		Ctrl (W1118) + PMA	1.22	0.10			24	12
E	eEPSC amplitude [nA]	Unc13-C1(HK) - PMA	20.26	2.25	Unpaired t-test	0.346400	17	9
		Unc13-C1(HK) + PMA	23.25	2.15			21	11
	PPR 10 ms ISI [nA]	Unc13-C1(HK) - PMA	1.09	0.13	Unpaired t-test	0.2943	17	9
		Unc13-C1(HK) + PMA	1.27	0.13			21	11

Table S18: Statistical information for experimental data from Figure S12. This table provides comprehensive statistical summaries for the experimental data illustrated in Fig.S12. It includes details on the tested variables, experimental groups, mean ± SEM values, the specific statistical tests employed, significance levels, and the total number of cells and animals analyzed.

Figure S12

	Measure	Group	Mean	SEM	Test Type	Significance level	n (animals)
A	Speed (mm/s)	Ctrl (W1118)	0.79	0.011	Unpaired t-test	1.00E-06	22
		Unc13-C1(HK)	0.55	0.01			22
B		Ctrl (W1118) - Fed	0.79	0.011	Paired t-test	9.50E-06	22
		Ctrl (W1118) - Starved	0.94	0.012			22
		Unc13-C1(HK) - Fed	0.55	0.01	Paired t-test	3.90E-06	22
		Unc13-C1(HK) - Starved	0.73	0.01			22
C		OK6-Gal4	0.605	0.0123	Unpaired t-test	0.052	21
		OK6>OAMB-RNAi	0.73	0.0175			23
D		OK6-Gal4 - Fed	0.605	0.0123	Paired t-test	0.0012	21
		OK6-Gal4 - Starved	0.825	0.0123			21
		OK6>OAMB-RNAi - Fed	0.73	0.0175	Paired t-test	0.016	23
		OK6>OAMB-RNAi - Starved	0.879	0.0128			23

Table S19: Statistical information for experimental data from Figure 6. This table provides comprehensive statistical summaries for the experimental data illustrated in Fig.6. It includes details on the tested variables, experimental groups, median ± SEM values, the specific statistical tests employed, significance levels, the total number of cells, animals, active zones and trajectories analyzed.

Figure 6

	Measure	Group	Median	SEM	Test Type	Statistical Test level	P-value	Significance	n (cells)	N (animals)	Azs	Trajectories
F	Median transformed diffusion coefficient (Log 10) ($\mu m^2/s$)	Unc13mEOS	-1.901	0.01057	Kolmogrov smirnof -test	0.03634		n.s.	10	6		5832
		Unc13mEOS-c1	-1.906	0.01902			0.0742		10	6		2306
G	Median ROC (µm)	Unc13mEOS	0.1395um	0.001035	Kolmogrov smirnof -test	0.02406		n.s.	10	6		5832
		Unc13mEOS-c1	0.14um	0.001677			0.3109		10	6		2306
H	Median Azdiameter (nm)	Unc13mEOS	68.27nm	1.894	Kolmogrov smirnof -test	0.1754		n.s.	10	6	108	
		Unc13mEOS-c1	70.34nm	2.591			0.0804		10	6	111	
J	Median Nc diameter (nm)	Unc13mEOS	32.45nm	1.079	Kolmogrov smirnof -test	0.1161		n.s.	10	6	108	
		Unc13mEOS-c1	30.37nm	1.064			0.4516		10	6	111	
I	Median AZ Density ($locs/nm^2$)	Unc13mEOS	0.1572	0.01282	Kolmogrov smirnof -test	0.3816		****	10	6	108	
		Unc13mEOS-c1	0.09566	0.00526			<0.0001		10	6	111	
K	Median NC Density (locs/nm2)	Unc13mEOS	0.06957	0.00611	Kolmogrov smirnof -test	0.4066		****	10	6	108	
		Unc13mEOS-c1	0.03857	0.002751			<0.0001		10	6	111	
P	Median transformed diffusion coefficient (Log 10) ($\mu m^2/s$)	Unc13mEOS-C1-IntCtrl	-1.828	0.03845	Kolmogrov smirnof -test	0.05881	0.8489	n.s	7	6		264
		Unc13mEOSC1-1MinOct	-1.82	0.05304					7	7		183
Q	Median ROC (µm)	Unc13mEOS-C1-IntCtrl	-1.82	0.1428	Kolmogrov smirnof -test	0.09426	0.2921	n.s.	7	6		264
		Unc13mEOSC1-1MinOct	0.1533	0.1533					7	7		183
R	Median Azdiameter (nm)	Unc13mEOS-C1-IntCtrl	78.33	78.33	Kolmogrov smirnof -test	0.1453	0.6884	n.s.	7	6	51	
		Unc13mEOSC1-1MinOct	74.48	74.48					7	7	55	
T	Median Nc diameter (nm)	Unc13mEOS-C1-IntCtrl	34.6	34.6	Kolmogrov smirnof -test	0.1066	0.9245	n.s.	7	6	51	
		Unc13mEOSC1-1MinOct	33.72	33.72					7	7	55	
S	Median AZ Density ($locs/nm^2$)	Unc13mEOS-C1-IntCtrl	0.01861	0.01861	Kolmogrov smirnof -test	0.2731	0.0548	n.s.	7	6	51	
		Unc13mEOSC1-1MinOct	0.02198	0.02198					7	7	55	
U	Median NC Density (locs/nm2)	Unc13mEOS-C1-IntCtrl	0.04535	0.04535	Kolmogrov smirnof -test	0.2624	0.0523	n.s.	7	6	51	
		Unc13mEOSC1-1MinOct	0.05546	0.05546					7	7	55	

Table S20: Statistical Information for Experimental Data from Figure S13. This table provides comprehensive statistical summaries for the experimental data illustrated in Fig.S13. It includes details on the tested variables, experimental groups, the specific statistical tests employed, significance levels, the total number of cells and animals analyzed.

Figure S13

	Measure	Group	Mean	SEM	Test Type	Significance level	n (cells)	N (animals)
C	Bleach Curve Analysis	Unc13mEOS	14.02	0.3814	t-test (unpaired)	1.26311E-07	33	2
		Unc13C1HK-mEOS3.2	10.92	0.3552			33	2

Table S21: Statistical Information for Experimental Data from Figure S14. This table provides comprehensive statistical summaries for the experimental data illustrated in Fig.S14. It includes details on the tested variables, experimental groups, median ± SEM values, the specific statistical tests employed, significance levels, the total number of cells, animals, active zones and trajectories analyzed.

Figure S14

	Measure	Group	Median	SEM	Test Type	Statistical test level	P-Value	Significance	n (cells)	N (animals)	Azs	Trajectories
E	Median transformed diffusion coefficient (Log 10) ($\mu m^2/s$)	Unc13mEOS -PhTx	-1.958	0.009747	Kolmogrov smirnof -test	0.06204	<0.0001		46	7		5736
		Unc13mEOS +PhTx	-2.028	0.01451				****	35	7		2568
F	Median ROC (µm)	Unc13mEOS -PhTx	0.1272	0.001051	Kolmogrov smirnof -test	0.06879	<0.0001		46	7		5736
		Unc13mEOS +PhTx	0.1172	0.001472				****	35	7		2568
I	Median Azdiameter (nm)	Unc13mEOS -PhTx	129.1	3.088	Kolmogrov smirnof -test	0.2029	<0.0001		46	7	276	
		Unc13mEOS +PhTx	113.8	2.447				***	35	7	228	
J	Median Nc diameter (nm)	Unc13mEOS -PhTx	40.07	2.566	Kolmogrov smirnof -test	0.2732	<0.0001		46	7	276	
		Unc13mEOS +PhTx	27.89	1.282				****	35	7	228	
G	Median AZ Density ($locs/nm^2$)	Unc13mEOS -PhTx	0.02162	0.001195	Kolmogrov smirnof -test	0.3371	<0.0001		46	7	276	
		Unc13mEOS +PhTx	0.02503	0.001103				****	35	7	228	
H	Median NC Density (locs/nm2)	Unc13mEOS -PhTx	0.03202	0.001255	Kolmogrov smirnof -test	0.217	<0.0001		46	7	276	
		Unc13mEOS +PhTx	0.0371	0.001111				****	35	7	228	

Table S22: Statistical Information for Experimental Data from Figure 7. This table provides comprehensive statistical summaries for the experimental data illustrated in Fig.7. It includes details on the tested variables, experimental groups, mean ± SEM values, the specific statistical tests employed, significance levels, and the total number of cells and animals analyzed.

Figure 7

	Measure	Group	Mean	SEM	Test Type	Significance level	n (cells)	N (animals)
A	mEPSP amplitude [mV]	UAS-OAMB-RNAi x W1118 -PhTx	0.8818	0.04629	Unpaired T-test	<0.0001	25	9
		UAS-OAMB-RNAi x W1118 +PhTx	0.5035	0.02144			24	8
	eEPSP amplitude [mV]	UAS-OAMB-RNAi x W1118 -PhTx	14.2	1.072	Unpaired T-test	0.1457	25	9
		UAS-OAMB-RNAi x W1118 +PhTx	12.03	0.9921			24	8
	Quantal content	UAS-OAMB-RNAi x W1118 -phTx	16.78	1.345	Unpaired test	0.047	25	9
		UAS-OAMB-RNAi x W1118 +PhTx	24.93	2.43			24	8
B	mEPSP amplitude [mV]	UAS-OAMB-RNAi x Ok6 -PhTx	0.952	0.04182	Unpaired T-test	<0.0001	24	8
		UAS-OAMB-RNAi x Ok6 +PhTx	0.4929	0.01603			19	7
	eEPSP amplitude [mV]	UAS-OAMB-RNAi x Ok6 -PhTx	21.16	1.751	Unpaired T-test	0.001	24	8
		UAS-OAMB-RNAi x Ok6 +PhTx	13.41	1.04			19	7
	Quantal content	UAS-OAMB-RNAi x Ok6 -PhTx	22.98	1.977	Unpaired T-test	0.1483	24	8
		UAS-OAMB-RNAi x Ok6 +PhTx	27.35	2.216			19	7

SI References

1. R. F. Laine *et al.*, NanoJ: a high-performance open-source super-resolution microscopy toolbox. *J Phys D Appl Phys* **52**, 163001 (2019).
2. I. Izeddin *et al.*, Wavelet analysis for single molecule localization microscopy. *Opt Express* **20**, 2081-2095 (2012).
3. N. Gustafsson *et al.*, Fast live-cell conventional fluorophore nanoscopy with ImageJ through super-resolution radial fluctuations. *Nat Commun* **7**, 12471 (2016).
4. F. Levet *et al.*, SR-Tesseler: a method to segment and quantify localization-based super-resolution microscopy data. *Nat Methods* **12**, 1065-1071 (2015).
5. M. Ovesný, P. Křížek, J. Borkovec, Z. Svindrych, G. M. Hagen, ThunderSTORM: a comprehensive ImageJ plug-in for PALM and STORM data analysis and super-resolution imaging. *Bioinformatics* **30**, 2389-2390 (2014).
